# Computational Approaches to Predict Hepatitis B Virus Capsid Protein Mutations That Confer Resistance to Capsid Assembly Modulators

**DOI:** 10.3390/v17030332

**Published:** 2025-02-27

**Authors:** Gideon Tolufashe, Usha Viswanathan, John Kulp, Ju-Tao Guo

**Affiliations:** Baruch S. Blumberg Institute, 3805 Old Easton Road, Doylestown, PA 18902, USA; gideon.tolufashe@bblumberg.org (G.T.); usha.viswana@gmail.com (U.V.);

**Keywords:** hepatitis B virus, capsid, capsid assembly modulators, drug resistance, molecular docking, MM/GBSA, molecular dynamics simulations

## Abstract

Capsid assembly modulators (CAMs) are a novel class of antiviral agents in clinical development for the treatment of chronic hepatitis B. CAMs inhibit hepatitis B virus (HBV) replication by binding to a hydrophobic pocket, i.e., HAP pocket, between HBV capsid protein (Cp) dimer–dimer interfaces to misdirect its assembly into empty capsids or aberrant structures and designated as CAM-E and CAM-A, respectively. Because the emergence of CAM-resistant variants results in the failure of antiviral therapy, it is important to rationally design CAMs with a high barrier of resistance for development. To establish computational approaches for the prediction of Cp mutations that confer resistance to CAMs, we investigated the interaction of representative CAM-A and CAM-E compounds, BAY 41-4109 and JNJ-56136379, with wild-type and 35 naturally occurring mutations of Cp residues at the HAP pocket using molecular docking, prime molecular mechanics with generalized Born and surface area solvation (MM/GBSA) and molecular dynamics (MD) simulation methods. Out of nine publicly available HBV capsid or CpY132A hexamer structures in the protein database, molecular docking correctly predicted the resistance and sensitivity of more than 50% Cp mutations to JNJ-56136379 with structures 5D7Y and 5T2P-FA. MM/GBSA correctly predicted the resistance and sensitivity of more than 50% Cp mutations to BAY41-4109 with the structures 5E0I-BC and 5WRE-FA, and to JNJ-56136379 with the 5E0I-FA structure. Our work indicates that only the capsid or CpY132A hexamer structure bound with a CAM with similar chemical scaffold can be used for more accurately predicting the resistance and sensitivity of Cp mutations to a CAM molecule under investigation by molecular docking and/or MM/GBSA methods.

## 1. Introduction

Hepatitis B virus (HBV) chronically infects 296 million people worldwide and causes more than 820,000 deaths annually due to cirrhosis and hepatocellular carcinoma (HCC) [1]. The current standard-of-care medications for chronic hepatitis B (CHB) include nucleos(t)ide analog (NA) HBV DNA polymerase inhibitors and pegylated alpha-interferon (Peg-IFN-α). While NA therapy can efficiently suppress HBV replication, reduce the viral load and prevent liver disease progression in the majority of treated patients, it fails to induce the loss of HBV surface antigen (HBsAg), an indication of durable immune control of residual HBV infection, i.e., the “functional” cure of CHB [2,3]. On the contrary, Peg-IFN-α therapy can induce HBsAg seroclearance in approximately 5% of treated patients, but its low therapeutic efficacy and poor tolerability limit its use [4]. Apparently, antiviral agents that can induce a functional cure in most treated patients are urgently needed.

HBV replicates its genomic DNA by packaging viral pre-genomic RNA (pgRNA) and DNA polymerase into capsids where the pgRNA is reverse-transcribed into a partially double-stranded, relaxed circular DNA (rcDNA) [5]. The genotype D HBV core (capsid) protein (Cp) is a 183-amino acid (aa) polypeptide containing an N-terminal assembly domain (NTD, aa 1–140) and an arginine-rich C-terminal domain (CTD, aa150–183). The assembly domain has five α helices connected by loops. The hydrophobic interaction between the α3 and α4 helices of two Cp monomers drives the formation of a four-helix bundle at their interface and results in the formation of Cp dimers that serve as the building block of capsids [6]. The assembly of 120 Cp dimers into an icosahedral capsid is primarily driven by the hydrophobic interaction between Cp dimer–dimer interfaces [7]. In the past two decades, multiple chemotypes of small molecules have been discovered to inhibit the packaging of the pgRNA–polymerase complex into capsids and consequentially prevent viral genome replication [8]. Mechanistic studies revealed that those compounds bind to a hydrophobic pocket, i.e., a HAP pocket, between the Cp dimer–dimer interface to misdirect the assembly of Cp dimers and are thus designated as capsid assembly modulators (CAMs) or core protein allosteric modulators (CpAMs) [9,10]. The HAP pocket is formed by approximately 29 amino acid residues of two monomers from each Cp dimer [11]. Due to the unique binding pose and specific interactions with different Cp residues at the HAP pocket, different CAMs modulate capsid assembly in slightly different ways. Based on the structural features of induced Cp dimer assembly products, CAMs can be classified into CAM-A compounds, such as heteroaryldihydropyrimidines (HAP), that misdirect Cp dimers to assemble aberrant capsids or non-capsid Cp polymers [12,13,14], and CAM-E compounds, such as phenylpropenamides (PPAs), sulfamoylbenzamides (SBAs), sulfamoylpyrrolamides (SPAs), and glyoxamoylpyrroloxamides (GLPs), that induce the Cp dimers to assemble morphologically “normal” capsids devoid of viral pgRNA and DNA polymerase, i.e., empty capsids [15,16,17,18,19,20,21,22,23].

Interestingly, CAM binding of assembled capsids induces significant structural changes in the HAP pocket and triggers overall and global structural alterations in Cp dimer, tetramer and capsids. For example, HAP1 binding of the HAP pocket in the context of a capsid moves Cp subunits and results in the fivefold vertices protruding from the bound capsid, the threefold vertices opening and the quasi-sixfold vertices flattening [24]. The HAP18 binding of capsids causes only minor changes in the quaternary structure and decreases the capsid diameter by ~3 Å. On the contrary, the AT-130 binding of capsids induces tertiary and quaternary structural changes and increases the capsid diameter by ~3 Å [25]. Interestingly, cryo-electron microscopy (Cryo-EM) reconstructions of preformed capsids treated with DBT1, a dihydrodibenzothiazepine, revealed that DBT1 binds to each of the four quasi-equivalent Cp interfaces, which is distinct from other CAMs that only bind to the HAP pocket formed between B and C Cp subunits [26]. Moreover, CAM binding of Cp dimer–dimer interfaces can also be analyzed by the crystallography of CpY132A heximers co-crystalized with different CAM molecules. Compared to a cryo-EM analysis of CAM-bound capsids that mostly revealed that CAM induced Cp tertiary and quaternary structural changes, the crystallography of CpY132A heximers revealed a more detailed interaction of the CAM molecule with amino acid residues of Cp at the HAP pocket due to an improved resolution [9,10].

More than ten CAMs have thus far been advanced to phase I or phase II clinical trials for the treatment of CHB and demonstrated potent antiviral activity [6,27]. However, as for other direct-acting antivirals, the emergence of CAM-resistant variants has resulted in the failure of CAM antiviral therapy in recent clinical trials [28,29]. Therefore, the development of computational methods to accurately predict Cp mutations that confer resistance to CAMs shall support the rational design and prioritization of CAMs with highly resistant barriers for further development. In our initial efforts to achieve this goal, we compared three computational chemistry methods for their ability to predict a panel of 35 naturally occurring Cp mutations that may confer resistance to representative CAM-A and CAM-E compounds with all the crystal or CryoEM structures of CAM-bound HBV capsids or CpY132 hexamers available in the protein database. Our study reported herein clearly demonstrates that no single computational chemistry method is universally suitable for the accurate prediction of CAM-resistant Cp mutations. Instead, due to the unique interaction of different chemotypes of CAMs with Cp at the HAP pocket, the capsid or Cp hexamer structure bound with a CAM of a similar chemical scaffold should be used for more accurately predicting the resistance and sensitivity of Cp mutations to a CAM molecule under investigation. In other words, if a novel chemotype of CAMs is under development, structures of representative compounds of this chemotype in complex with an HBV capsid or CpY132A hexamers should be resolved for a structure-guided structure–activity relationship analysis and the prediction of drug-resistant Cp mutations.

## 2. Materials and Methods

### 2.1. Structure Preparation

HBV capsid or CpY132A hexamer structures with the accession codes 5D7Y, 4G93, 5E0I, 5WRE, 5T2P and 6WFS were used in this study (Table 1). These structures were prepared for docking using the protein preparation protocol in Maestro to add missing hydrogens, assign the proper protonation states and perform minimization to avoid bad atom contacts. 5D7Y, 4G93 and 6WFS are structures of the HBV capsid bound by HAP18 [30], AT-130 [25] and DBT1 [26], respectively. 5E0I and 5WRE are CpY132A hexamers bound with NVR-010-001-E2 [9] and HAP_R01 [10], respectively. 5T2P is a crystal structure of the CpY132A hexamer bound with SBA_R01 [10]. While HAP18, NVR-010-001-E2 and HAP_R01 are structurally similar CAM-A compounds, AT-130, SBA_R01 and DBT1 are structurally distinct CAM-E compounds (Figure S1).

**Figure 1:**
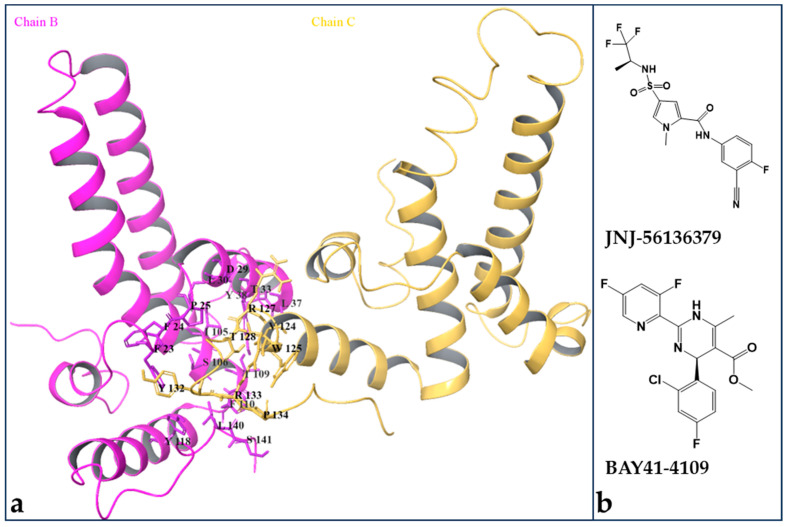
The 3D structure of the HBV Cp tetramer and the 2D structure of the CAM compounds used in this study. (**a**) The residues at the interface of the two subunits of two Cp dimers in the context of the HBV capsid (PDB code: 5D7Y), i.e., the HAP pocket, are highlighted. (**b**) The 2D structures of JNJ-56136379 and BAY41-4109.

Because different chemotypes of CAMs, such as HAP, SBA, PPA and DBT, uniquely bind to the HAP pocket at the interface between different quasi-equivalent Cp subunits and induce distinct allosteric changes in Cp dimers, tetramers and capsids as discussed above, it is not surprising that different CAMs have common and distinct drug resistance profiles [11,31]. Because we do not know which structure system is better suited for drug-resistant prediction, thirty-five HBV Cp single amino acid substitutions representing the most common naturally occurring Cp mutations of residues around the HAP pocket (Figure 1a) were made for all the structures reported in Table 1 and subjected to docking, MMGBSA calculations and molecular dynamics (MD) simulations for JNJ-56136379 and BAY41-4109, representative CAM-E and CAM-A compounds, respectively (Figure 1b). The reason for choosing those two CAMs for computational drug-resistant prediction studies is because their activities against HBV with wildtype and all thirty-five mutant capsid proteins used in this study had been determined in HBV replicon plasmid-transfected HepG2 cells [32].

We used the Glide program embedded in Schrodinger2023-1 software for docking CAMs into the hydrophobic pocket of different structures of CAMs bound to the HBV core protein (Table 1). BAY41-4109 and JNJ-56136379 were docked into the HAP pocket of the wildtype and mutant structures. Thereafter, we used the Prime MM-GBSA tool to calculate the free energy of the docked complexes, allowing protein flexibility and adding an implicit water model in the simulation. Finally, molecular dynamics simulations (MD) for 150 ns were run on a few selected systems on the AMBER 18 program to investigate the dynamics and energy of the systems within the stipulated time frame. The binding free energies obtained from the docking process, prime MM-GBSA and MD programs for the wildtype and mutant Cps were compared to rank mutations that are sensitive or resistant across different structures tested.

### 2.2. Molecular Docking Calculations

All nine structures (wildtype and 35 Cp mutations) had their energy minimized with the OPLS4 forcefield [33] using the MacroModel-v13.4 module on Schrodinger2023-1 [34]. The 2D structures of compounds JNJ-56136379 and BAY41-4109 (Figure 1b) were drawn and converted to 3D low-energy structures using the LigPrep module [35]. First, the bound CAMs in the different structures were docked into the binding pocket to ascertain the reproducibility of the docking algorithm and reliability in testing new compounds. The prepared JNJ-56136379 and BAY41-4109 structures were docked into the binding pocket of the HBV capsid using the Glide module. Docking poses and interactions were displayed using the 2D ligand interaction diagram on Maestro. The docking scores of CAM-HBV complexes were computed using the Emodel scoring function to rank compounds that bind strongly [36].

### 2.3. Post-Docking Prime MM/GBSA Calculations

The relative binding energy of a ligand (CAM) in complex with the wildtype or mutants of the HBV capsid or CpY132A hexamer was calculated using the prime MM/GBSA module in Schrödinger Maestro version 13.1 as a post-docking validation. Binding free energies of compounds have been shown to correlate reasonably with ranking based on the experimental binding affinity with structurally similar compounds [37]. The MM/GBSA method applies the variable-dielectric generalized Born (VSGB) solvation model [38] to compute the electrostatic component of the solvation binding energies. The prime MM/GBSA accounts for protein flexibility; thus, the distance from the ligand is defined as 5.0 Å to the protein flexible residues. The binding energy of the receptor and ligand is computed as

∆*G_bind_* = ∆*G*_complex_ − ∆*G*_ligand_ − ∆*G*_receptor_ (1)

In Equation (1), ∆*G*_complex_ is the absolute free energy of the complex, ∆*G*_receptor_ is the absolute free energy of the protein and ∆*G*_ligand_ is the absolute free energy of JNJ-56136379 or BAY41-4109. The individual components of ∆*G*_*bind*_ are defined by

∆*G_bind_* = ∆*E_MM_* + ∆*G_solv_* − ∆*G*_SA_ (2)

where ∆*E*_*MM*_ is the difference in energy between the complex and the sum of the energies of the receptor with and without a ligand, ∆*G*_*solv*_ is the difference in the GBSA solvation energy of the complex and the sum of the solvation energies for the ligand and unliganded protein, and ∆*G*_SA_ is the difference in the surface area energy for the complex and the sum of the surface area energies for the ligand–receptor complex.

### 2.4. Molecular Dynamics Simulation Calculations

To account for conformational changes during ligand–protein interactions in a solvated environment, we carried out molecular dynamics (MD) simulations of selected complexes of wildtype and mutants of Cp T33N, T33S and T33P, which confers high drug resistance to several different chemotypes of CAMs [11,28,31,39]. The best docked pose of the two compounds (JNJ-56136379 and BAY41-4109) at the dimer–dimer interface showing interaction with Trp102 was used as the starting structure for the MD calculations. Using the Leap module on AMBER18, missing hydrogen atoms were added to the complexes [40]. The AMBER force field 14SB [41] and the general AMBER force field (GAFF) [40] were used to define the proteins and inhibitors, respectively. The complexes were neutralized by adding the required number of ions (12.0 Na+) before solvation. The complexes were solvated in a truncated octahedral cell of TIP3P water molecules [42], extending 12 Å outside the protein on each side; thereafter, the parameter and topology files were saved for molecular dynamics simulations. The two minimization steps were performed using 5000 frames of steepest decent minimization followed by 10,000 frames of conjugated gradient minimization to remove the overlapping of atoms. Afterwards, the minimized systems were heated from 0 to 300 K with solute restrained at 300 ps, and then 50 ps of density equilibration was applied with weak restraints on solutes and 2000 ps of constant pressure equilibration at 300 K. A total of 150 ns MD simulations for each wildtype and mutants in complex with JNJ-56136379 and BAY41-4109 were performed at a constant temperature of 300 K and a constant pressure of 1 atm using the GPU version of PMEMD [43]. The time step of 2 fs was used for all simulations. The MD trajectories were analyzed using the CPPTRAJ module implemented in AMBER18 software [44].

## 3. Results and Discussion

HBV capsid assembly is primarily driven by the hydrophobic interactions at Cp dimer–dimer interfaces. CAMs misdirect the assembly of Cp dimers into empty capsids or altered structures by binding to the hydrophobic pocket between Cp dimer–dimer interfaces to accelerate the kinetics and/or alter the pathway of the assembly process. Not surprisingly, many single amino acid substitutions of HBV Cp residues at the Cp dimer–dimer interface have been demonstrated to interfere with capsid assembly, pregenomic RNA (pgRNA) packaging, viral DNA synthesis and virion particle production [10,11,45]. Moreover, some of those Cp mutations do not significantly reduce the fitness of HBV replication in hepatoma cells but confer resistance to one or multiple chemotypes of CAMs [11,28,31,46]. Because the currently available HBV infection cell culture systems cannot support multiple rounds of HBV infection, CAM-resistant HBV variants cannot be experimentally selected and validated as for many other viruses. Instead, drug-resistant profiles of CAMs are currently determined by cell-based antiviral assays against a large panel of Cp mutant HBV replicon constructs in transfected human hepatoma cells. This technical limitation makes drug-resistant profiling during the lead optimization of CAMs very difficult. To overcome this limitation, we intend to develop a computation chemistry approach to predict the drug resistant profile and facilitate CAM lead optimization efforts. Naturally occurring HBV Cp mutations observed in chronic HBV carriers are generally replication competent and thus can be selected as CAM-resistant HBV variants under CAM therapy. Accordingly, a panel of 35 naturally occurring single amino acid substitutions of residues around the HAP pocket were selected to predict their sensitivity or resistance to BAY41-4109 or JNJ-56136379 by using three computational methods with the workflow illustrated in Figure S2. The predicted drug resistance will be compared with the fold of resistance (relative resistance) of those drugs obtained from antiviral assays in HBV replicon-transfected human hepatoma cells [32]. A Cp mutation that confers equal or more than a twofold increase in EC_50_ to a CAM compound compared to that of a wildtype HBV replicon is defined as a drug-resistant mutation in this study.

### 3.1. Comparative Analysis of CAM Binding and Induced Allosteric Changes in HBV Capsid Protein

For a deep analysis of CAM interaction with Cp at the dimer–dimer interface and the optimal prediction of Cp mutations that confer resistance to CAMs, we analyzed CAM interaction with Cp in the context of capsids or CpY132A hexamer from the structures deposited in the database in detail. 5D7Y (HAP18), 6WFS (DBT1) and 4G93 (AT130) structures have four quasi-equivalent subunits containing chains A to D as capsids, while 5E0I (NVR10_001E2), 5WRE (HAP_R01) and 5T2P (SBA_R01) have six quasi-equivalent subunits containing chains A to E as CpY132A hexamers. The HBV Cp binding site comprises a pocket in one subunit that is capped by residues from the neighboring subunit. CAMs bind at the interfaces of subunits (for example, B and C) with strong density and similar strength to other subunits.

**Figure 2:**
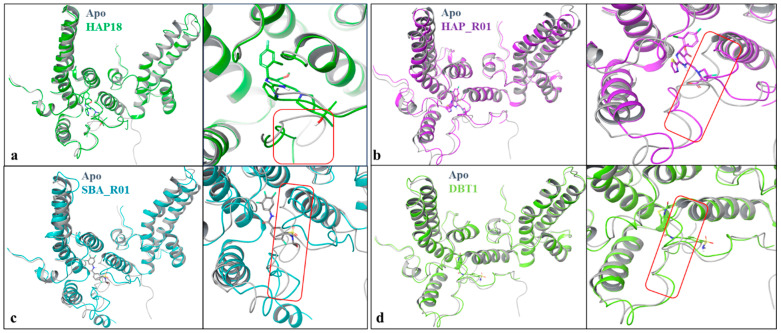
An overlay of some CAM structures onto the apo structure (3J2V, gray) reveals quaternary and local structural changes near the ligand binding sites. (**a**–**d**) There are significant differences in structure, as all the CAMs induce conformational changes in the hydrophobic pocket, indicative of opening to accommodate compound binding compared to the close apo structure, shown in the region marked in red.

First, significant structural changes are induced in the Cp monomer and dimer–dimer interface in the context of the HAP18 and DBT1 binding of the HBV capsid or the SBA_R01 and HAP_R01 binding of the CpY132A hexamer (Figure 2). There are minimal global conformational changes between bound HAP18 in comparison to the apo structure. However, the C terminal tail shifts by up to 4 Å away from the apo, as shown in Figure 2, creating room to accommodate the ligand in the pocket and thus being suggestive of lock-and-key binding to accompany the observed shift in the capping C subunit. A significant shift is observed for HAP_R01 (highlighted in red), with helix 5 moving away compared to the apo structure. For bound SBA_R01 in comparison to the apo structure, there are specific structural changes, particularly in helix 5, and the turn (residues 126 to 135), the loop region shifts by up to 3 Å compared to the apo structure to create a binding pocket for SBA_R01. DBT1 fits into the pocket, wrapping around helix 5 from the neighboring capping subunit with noticeable structural changes.

**Figure 3:**
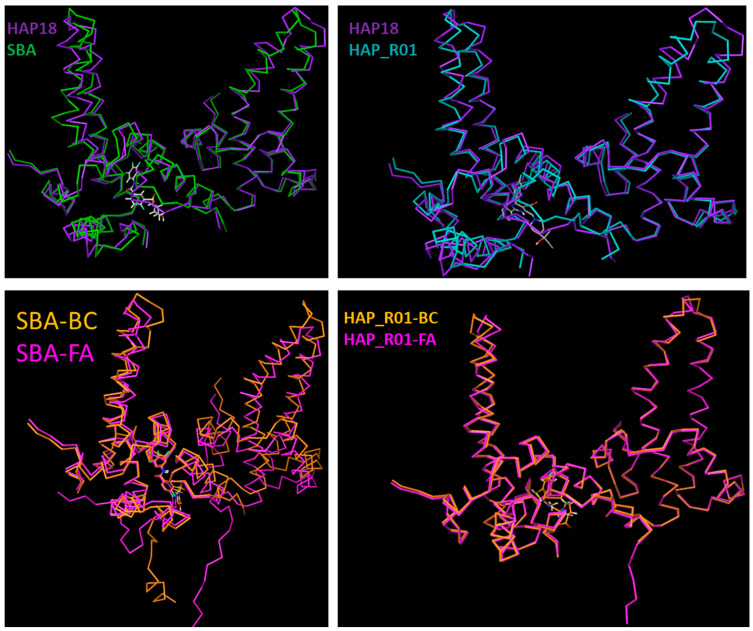
A comparison of HAP- and SBA-bound crystal structures showing distinctly different changes in tertiary and quaternary structures at the level of the dimer. Despite similar HAP/SBA binding sites being occupied, the effect on the local structure is distinct in each case. (**Top panels**) The overlay of BC dimers from the HAP18-bound capsid structure (5D7Y, purple) on corresponding subunits from the SBA-bound capsid structure (5T2P, green) based on the superposition of capsid symmetric units. There are significant differences in structure as both HAP and SBA induce conformational changes affecting the binding pocket and the global structure. (**Bottom panels**) The overlay of BC and FA dimers from the SBA-bound capsid structure (5T2P, orange (BC) magenta [FA]) on corresponding subunits from the HAP_R01-bound capsid structure (5WRE, orange (BC) magenta [FA]). There are large differences in the tertiary structure, especially in the SBA-bound structure.

Significant differences exist in the bound HBV capsid structures as both HAP and SBA induce conformational changes affecting the binding pocket (Figure 2) and the tertiary/quaternary structure, as shown in Figure 3. There are large differences in the tertiary structure, especially in the SBA/HAP bound structure between different dimers of 5T2P and 5WRE (Figure 3).

**Figure 4:**
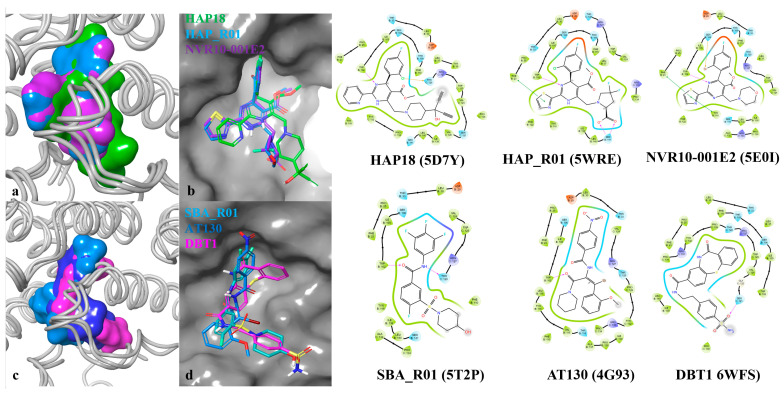
Comparison of HAP (**a**,**b**) and other CAM (**c**,**d**) compounds at dimer–dimer interface and ligand–protein interactions of selected CAMs bound to HBV capsid and hexamer structures, as observed in X-ray or CryoEM.

Comparing the interactions of CAMs to Cp residues at dimer–dimer interfaces, HAP compounds made hydrogen bonds with the dihydropyrimidine at the 3-nitrogen of HAP18, HAP_R01 and NVR10-001E2, while the benzamide oxygen of SBA_R01 and the piperidine oxygen of DBT1 acted as hydrogen bond acceptors from TRP102. The thiazole of HAP_R01 and NVR10-001E2 made a Pi–Pi stacking interaction with PHE23, while the pyridine of HAP18 was unproductive but space-filling (Figure 4a–d). Ligand surfaces in the pocket shown in Figure 4a,c reveal that HAP18 and DBT1 protrude more to the solvent-exposed area. The carboxylic group in HAP_R01 made hydrogen bonding interactions with SER141 through its acceptor and donor atoms’ capabilities. At the same time, the R-group at the same position for HAP18 and NVR10-001E2 tends to cause steric hindrance and occupy space. Other interactions include halogen bonds with THR128 (for HAP18), SER106 for NVR10-001E2 and a pi bond with HAP_R01. These interactions are key to predicting both the resistance and sensitivity of compounds to HBV mutations. Thus, these interactions should be maintained with sensitive mutants, while the loss of interactions should be observed for resistant mutants.

Measuring the binding site surfaces of Cp dimers of bound HBV capsid and hexamer structures revealed differences in the pocket volume based on the occupancy of the compounds, as presented in Table 2. Specifically, an increase in the molecular weight (SBA_R01 < DBT1 < AT130 < NVR10-001E2 < HAP_R01 < HAP18) of the compounds led to an increased ligand surface area. The receptor surface area created upon the ligand binding of the commonly bound BC chain (via the dimer–dimer interface) of capsid differs from the chain FA interface of the hexamer structure (via the dimer–dimer interface), indicating that the Cp dimer–dimer interface of FA is larger than that of BC. We therefore inferred that a Cp dimer with a larger volume might be suitable for mutational studies due to some amino acids with bulky side chains.

### 3.2. Molecular Docking

**Figure 5:**
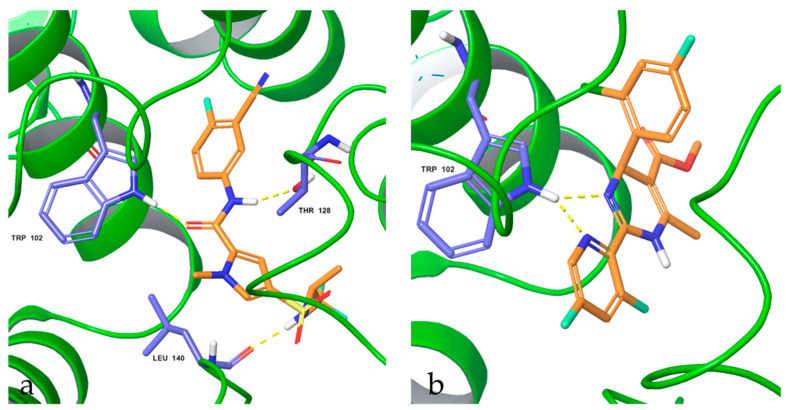
Docking poses and interaction of (**a**) JNJ-56136379 and (**b**) BAY41-4109 with wildtype capsid protein. Residues shown are within 5Å, having interactions with JNJ-56136379 and BAY41-4109 (in orange). Hydrogen bonds are shown in yellow. Similar conformations were obtained with other mutants; see supplementary information.

For some of the mutated amino acid residues, the default and most populated rotamers which favor nucleophilic interaction were selected, and the default rotamer was the most populated empirically determined conformation. Thereafter, the structures had their energy minimized to obtain a low energy state in the energy landscape. Due to the structural difference between these two compounds (JNJ-56136379 and BAY41-4109), we modeled the interactions as shown in Figure 5. JNJ-56136379 binds to the wildtype Cp with three hydrogen bonding interactions: the hydrogen bond contact between the amide carbonyl oxygen atom and the NH group of the indole ring of Trp102, the hydrogen bond between the amide NH group with the side chain oxygen atom of Thr128, and the hydrogen bond of the NH group to the hydroxyl of Leu140 (Figure 5a). Two hydrogen bonds of the NH group of the indole ring of Trp102 bind to BAY41-4109 (Figure 5b).

The results of the docking calculations performed using the Glide scoring function observed for the JNJ-56136379 and BAY41-4109 compounds are summarized in Table 3 for the top predictive systems (at least 50% prediction for both resistant [lower docking scores than the WT] and sensitive mutants [higher docking scores than the WT]) and Tables S1–S9. Based on the docking scores in comparison to the wildtype, some variants altered the binding pattern and reduced the binding affinity. Using the crystal structures of the HBV capsid in complex with HAP (5D7Y, chain BC) and SBA (5T2P, chain FA) gave the best prediction of both resistance and sensitivity to JNJ-56136379 and BAY41-4109 (Table S10). For JNJ-56136379, comparing the docking score of the wildtype and other Cp mutants, 8/16 (50%) resistant mutants (i.e., relative resistance ≥ 2) were predicted to show reduced binding scores, while 10/15 (67%) resistant mutants showed reduced binding scores for BAY41-4109. In addition, 16/18 (89%) Cp mutants sensitive (i.e., relative resistance ≤ 2) to JNJ-56136397 showed similar or increased binding scores with the WT Cp, whereas 8/19 (45%) Cp mutants sensitive to Bay 41-4109 showed similar or increased binding scores to the WT Cp. In summary, HAP18/5D7Y predicted favorably for JNJ-56136379 and the SBA_R01/5T2P-FA model predicted resistant/sensitive mutants for both JNJ-56136379 and BAY41-4109. Furthermore, SBA_R01 and JNJ5613-6379 have similar structural features to the SBA scaffold and thus favor its prediction (Table 3).

**Figure 6:**
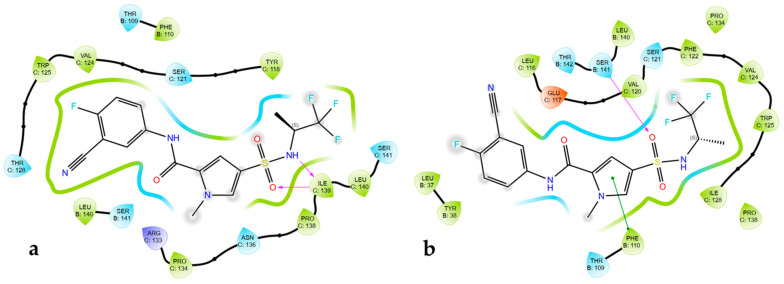
Ligand interaction from docking model of JNJ-56136379 with HBV capsid protein (PDB ID: 5D7Y). (**a**) T33N and (**b**) T128I mutation interaction; hydrogen bonds are shown in pink, while green is pi stacking bond.

Some of the studied mutants at the core protein binding pocket showed reduced or improved activity to JNJ-56136379 and BAY41-4109 as revealed by a cell-based antiviral assay [32]. Using the docking score, resistance to JNJ-56136379 was predicted for P25A, T33N, L37Q, S106T, F110I, Y118F, V124G and T128I; it was also accurately predicted that JNJ-56136379 was sensitive to mutants such as D29H, Y38F, Y38H, I105L, I105V, T109A, T109I, T109M, T109S, R133K, L140I and S131P. Notably, some resistant mutants lost interactions with important residues such as W102, L140, and S141, while sensitive mutants maintained key interactions with new ones formed. Similarly, BAY41-4109 was predicted for resistant mutants such as F23Y, F24L, P25G, P25S, D29G, T33S, T33N, L37Q, T109M, T109S and R127 as well as sensitive single amino acid substitutions like S106T, T109A, T109S, F110I, T128I, R133K and S141P. To justify the rationale behind these resistant mutants (T33N and T128I) to JNJ-56136379 with an HBV capsid, we modeled 5D7Y/HAP18. The mutation of T33N lost critical hydrophobic interactions, while T128I made polar contact with residues F110 and S141, which are within the list of mutations reducing JNJ-56136379 activity (Figure 6). The docking model of BAY41-4109 with the Cp Y132A mutant in complex with the sulfamoylbenzamide (SBA_R01) structure predicted resistant mutants T33N and P25S. T33N and P25S lost critical hydrophobic interactions upon binding to BAY41-4109 (Figure S3).

**Figure 7:**
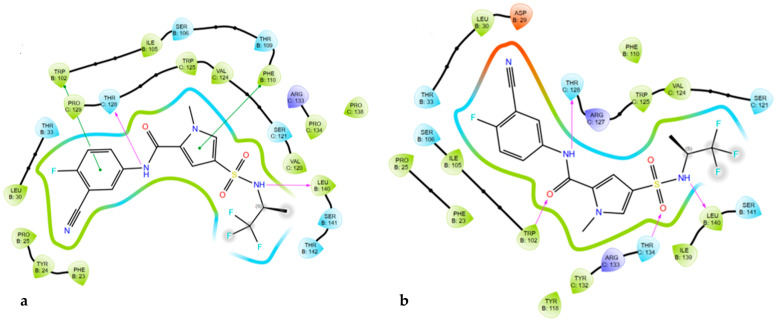
Ligand interaction from docking model of JNJ-56136379 with HBV capsid protein (PDB ID: 5D7Y). (**a**) F24Y and (**b**) P134T mutation interaction; hydrogen bonds are shown in pink, while green is pi stacking bond.

Similarly, selected sensitive mutants to JNJ-56136379 such as F24Y and P134T (Figure 7) maintained important interactions with T128 and W102 with additional interactions with F110 and L140. Mutation P134T interacted with the mutated residue T134, increasing JNJ56136379 activity.

Some selected sensitive mutants to BAY41-4109, like F110I and T128I, showed pi stacking and hydrogen bond interactions with W102, while T128I made polar contact residues with F110; these residues are within the list of mutations increasing BAY41-4109 activity (Figure S4).

The docking of JNJ-56136379 and BAY41-4109 to HBV capsid protein/mutants can reproduce the experimental pose observed for HAP and SPA moieties in the HBV T4 capsid using a crystal structure bound with a similar chemotype for the best prediction efficiency (Figure S5). Since receptor backbone flexibility and the movement of several key secondary elements of the receptor involving ligand binding is still a major challenge in docking studies to predict binding energies accurately [47], there is a need to utilize the prime MM/GBSA method, which can deal with side-chain flexibility and has been proven effective in calculating the binding energies of ligand–protein complexes.

### 3.3. Post-Docking Prime MM/GBSA

The interaction between the wildtype and mutants of the HBV capsid protein and JNJ-56136379/BAY41-4109 compounds in all complexes, as well as the influence of protein flexibility and solvent effects in the HAP pocket, was evaluated using MM/GBSA were obtained. This approach gives a more reliable score with is comparable to experimental affinity, i.e., the more negative value of MM/GBSA binding energies indicates a stronger binding of the ligand-receptor complex.

The free energy of binding (∆G_bind_) for the given complexes varied from −102.10 to −75.23 kcal/mol (Table S11). Using the crystal structure of the HBV capsid in complex with HAP (5E0I, chain BC) gave the best prediction of resistance and sensitivity to JNJ-56136379 and BAY41-4109. The MM/GBSA scores for the wildtype were -84.4 kcal/mol and −97.62 kcal/mol for JNJ-56136379 and BAY41-4109, respectively. For JNJ-56136379, comparing the ΔG from MM/GBSA of the wildtype and other Cp mutants, 14/16 (88%) resistant mutants (i.e., relative resistance ≥ 2) were predicted to show reduced binding scores, while 9/15 (60%) resistant mutants showed reduced binding scores for BAY41-4109. In addition, 7/17 (41%) Cp mutants sensitive (i.e., relative resistance ≤ 2) to JNJ-56136397 showed similar or increased binding scores with WT Cp whereas 10/20 (50%) Cp mutants sensitive to BAY41-4109 showed similar or increased binding scores to WT Cp. When comparing the different Cp dimers, it is obvious that chain FA has a bigger volume than chain BC (Table 2) which could accommodate different amino acid side chains. Models 5T2P and 5WRE, both chains FA which are hexameric structures predict both sensitive and resistant mutants (Table S11). Overall, the MMGBSA approach yields favorable predictions of both the sensitive and resistant mutants against JNJ-56136379 and BAY41-4109 (Table 4).

Many of the mutants showed reduced binding in comparison to the wildtype with BAY41-4109, while significant reductions were observed for most of the mutants with JNJ-56136379. This clearly shows that alteration occurred in the binding pattern and can be better revealed with the MM/GBSA calculations compared to rigid docking. Most importantly, the MMGBSA approach accurately predicted resistance to the naturally occurring amino acid substitution at the core amino acid position 33 (T33N, T33P and T33S), part of the CAM-binding pocket for both compounds. As expected, the binding energies calculated using the prime MM/GBSA approach perform better than the docking method, especially for highly resistant T33 substitutions. The MMGBSA score tends to overestimate binding free energies as the distance from the ligand is increased, allowing for more protein flexibility. For these systems, the MM/GBSA method performs better than the docking method; however, it predicts favorably for resistant mutants. Thus, we employed real-time MD simulations and thereafter calculated the binding energy after 150 ns. The prime MM/GBSA still suffers setbacks since it cannot predict how every atom in a protein or other molecular system will move over time based on a general model of the physics governing interatomic interactions [48]; thus, there is a need to use a new method called molecular dynamics simulations.

### 3.4. Post-MD Simulations and Binding Free Energy Calculations

HAP derivatives bound with the HBV capsid have been reported to differentially affect assembly and increase the stability of protein–protein interactions [49]. Due to the computational demands of MD simulations, the interaction between the wildtype (HAP18 bound to HBV capsid 5D7Y, which showed a favorable prediction of resistance/sensitivity from docking and MMGBSA) and some selected mutants of the HBV capsid dimer and JNJ-56136379 and BAY41-4109, in addition to their structural and dynamical properties at the HAP pocket, were investigated using MD simulations. Also, an MD simulation can deal with the flexibility of both the ligand and protein more effectively than other algorithms [47]. The theoretical binding free energies were calculated from the last 10 ns of the MD simulation interval with explicit water molecules as the solvent for some selected resistant and sensitive mutants. The binding free energies were computed using MM/GBSA analysis from 1000 frames for all the complexes and are presented in Tables S12 and S13.

It was predicted that ΔG_bind_ of JNJ-56136379 (Table S12) and BAY41-4109 (Table S13) was in complex with the wildtype-resistant mutants F23Y, P25G, T33N, T3P, T33S, L37Q, R110I and Y118F and sensitive mutants such as D29H, Y38H, I105L, L140I and P134T. The calculated values of the binding free energies follow the same trend with the relative resistance for these mutants determined experimentally [32]. Importantly, as expected, the binding free energies of JNJ-56136379 and BAY41-4109 bound with the wildtype are higher than the mutants suggestive of the reduced affinity in the presence of mutations. Other important energy components are listed in Tables S12 and S13 for all complexes. The binding is dominated by van der Waals (ΔE_vdw_) interactions, consistent with the hydrophobicity of the binding pocket, as depicted by the ΔE_vdw_ values with more negative energies. In addition, the nonpolar contributions (ΔG_nonpolar_, i.e., more negative) for the complexes are stronger than the polar contributions (ΔG_polar_), which further supports the hydrophobic nature of the HAP binding pocket critical to facilitate the HBV capsid assembly process [50].

Aside from hydrophobic interactions, another vital parameter is hydrogen bonds as a main driving force in drug–receptor interactions [51]. The hydrogen bonding interactions between JNJ-56136379 and BAY41-4109 and the HAP pocket residues and their percentage occupancy throughout the simulation time are listed in Table S14. For the wildtype, for 19% of the simulation time, there is a hydrogen bond between the nitrogen atom of the residue Trp102 and the nitrogen atom of atom #1 of BAY41-4109, while 51% of the time, we see a hydrogen bond between the nitrogen atom of the residue Trp102 and the oxygen atom of atom #3 of JNJ-56136379. In contrast, there is a decline in the percentage occupancy of hydrogen bonding in the variants studied, supporting the effects of mutations on the binding of these compounds. Noticeably, the hydrogen bonds are very short-lived, causing insignificant stability in the ligand–protein interaction. Other residues form hydrogen bonds with the ligand–protein complex, such as Leu140, Ser141 and Thr142.

As expected, the binding free energies from the MD method perform better than the docking and prime MM/GBSA. However, this method is still unable to predict accurately for all mutants, and thus, a combination of several methods is highly recommended.

### 3.5. Concluding Remarks

In summary, we first assessed the binding patterns of JNJ-56136379 and BAY41-4109 in the HAP binding pocket of the wildtype and 35 naturally occurring mutants of the HBV capsid dimer. These compounds bind at the same site with similar orientations, as seen with the wildtype bound with HAP18, but mutations at this site, change the interactions observed. Applying the docking method was not sufficient to predict how HBV capsid protein mutations confer resistance to CAMs based on the low resistance/sensitivity prediction of JNJ-56136379 and BAY41-4109. Then, with the introduction of protein flexibility and the solvation effect, the Glide MM/GBSA could give a further prediction. Furthermore, a molecular dynamics study was utilized to study the interaction of JNJ-56136379 and BAY41-4109 and the wildtype/selected mutants of the HBV capsid dimer. The free binding energies of these complexes were calculated from an MD simulation in explicit water molecules. The ΔG_bind_ of these compounds bound with the wildtype is greater than all mutants tested, suggesting the reliability of the MD simulation in correlating with the experimental results in terms of resistance but predicting less for sensitive mutants. However, we observed prevalent hydrogen bond interactions between TRP102 with the WT-JNJ-56136379 and WT-BAY41-4109 in comparison to the mutant complexes. Interactions between these compounds and the following residues are important: Ser106, Tyr118, Thr128, Leu140 and Ser141. Overall, using the wildtype structure bound with the HAP chemotype does not provide a good prediction for other chemotypes such as SPA due to differences in their sizes, which created variations in the binding pocket where the compound can accommodate; thus, one ligand conformation is not suitable for mutational studies. Therefore, the use of different CAM-bound crystal structures for mutation modeling revealed favorable predictions using similar chemotypes of HAP and SPA moieties in the HBV T4 capsid for the best prediction efficiency. In addition, Cp dimers with larger binding pockets (PDB: 5T2P and 5E0I) are highly recommended for mutational studies. Furthermore, our study indicates that a similar CAM-bound structure should be used for drug design purposes to enhance the accurate prediction of the resistance and sensitivity of Cp mutations to a CAM molecule. Although some of the high resistance mutants were still not predicted using the MD, this may be due to a few reasons such as poor crystal structures, the uncertainty of conformations/dynamics of available cryo-EM structures, and long-range conformational changes in the capsid structure that were not considered in the current study. Moreover, it should be noted that our structural analysis of CAMs with Cp dimers is based on the structures of assembled capsids or Cp132A hexamers, whereas the interaction of CAMs with Cp dimers occurs during the assembly of Cp dimers into capsids or hexamers. These obvious differences certainly limit the accuracy of the computation prediction of CAM activity for wildtype and mutant Cp assembly because the HBV capsid pocket moves upon ligand binding, impacting its pharmacological effects. In the future, we will explore long MD simulations on different conformations in addition to investigating the role of structure waters in ligand binding at the HAP pocket.

## Figures and Tables

**Table 1:** A list of structures used in this study.

Structure	Structure Accession No.	HAP Pocket Between Quasi-Equivalent Cp Subunits
**HAP**		
capsid with HAP18	5D7Y	BC
hexamer with HAP_R01	5WRE	BC
hexamer with HAP_R01	5WRE	FA
hexamer with NVR10-001E2	5E0I	BC
hexamer with NVR10-001E2	5E0I	FA
**SBA**		
hexamer with SBA_R01	5T2P	BC
hexamer with SBA_R01	5T2P	FA
**PPA** capsid with AT130	4G93	BC
**DBT**		
capsid with DBT1	6WFS	BC

**Table 2:** Binding site surfaces (unit in Å^2^) for HBV receptors and CpAMs truncated at 5.0 Å from each ligand.

PDB/Ligand Name	Ligand Area *	Receptor Area *
5T2P/SBA_R01	336.939 (351.654)	1107.602 (1942.785)
6WFS/DBT1	399.815	1635.339
4G93/AT130	385.11	1538.402
5E0I/NVR10-001E2	381.005 (381.132)	1239.273 (1919.374)
5WRE/HAP_R01	404.144 (406.959)	1474.02 (2037.358)
5D7Y/HAP18	462.417	1679.9

* Values in brackets are for chain FA surfaces.

**Table 3:** Quantification of prediction rates using docking (percent) with different structures.

Structure	Metric	JNJ-56136379 (%)	Overall Prediction Rate (%)	BAY41-4109 (%)	Overall Prediction Rate (%)
5D7Y	Sensitive	89 (16/18)	70.6	52 (10/19)	42.8
	Resistance	50 (8/16)		31 (5/16)	
4G93	Sensitive	38 (6/16)	37.5	67 (12/18)	51.5
	Resistance	38 (6/16)		33 (5/15)	
5E0I	Sensitive	28 (5/18)	50.0	25 (5/20)	51.4
	Resistance	75 (12/16)		87 (13/15)	
5E0I-FA	Sensitive	22 (4/18)	50.0	35 (7/20)	54.3
	Resistance	81 (13/16)		80 (12/15)	
5T2P-BC	Sensitive	6 (1/18)	44.1	32 (6/19)	44.1
	Resistance	88 (14/16)		60 (9/15)	
5T2P-FA	Sensitive	76 (13/17)	63.6	45 (8/19)	52.9
	Resistance	50 (8/16)		67 (10/15)	
5WRE-BC	Sensitive	39 (7/18)	46.8	6 (1/18)	34.3
	Resistance	57 (8/14)		71 (10/14)	
5WRE-FA	Sensitive	90 (18/20)	66.5	95 (19/20)	58.8
	Resistance	6 (1/15)		7 (1/14)	
6WFS-BC	Sensitive	28 (5/18)	51.4	35 (7/20)	37.1
	Resistance	81 (13/16)		40 (6/15)	

Highlighted in green are systems with at least 50% prediction of both resistance/sensitivity.

**Table 4:** Quantification of prediction rates using MMGBSA scores (percent) with different structures.

Structure	Metric	JNJ-56136379 (%)	Overall Prediction Rate (%)	BAY41-4109 (%)	Overall Prediction Rate (%)
5D7Y	Sensitive	56 (10/18)	50.0	0(0/19)	40.0
	Resistance	44 (7/16)		88 (14/16)	
4G93	Sensitive	75 (12/16)	46.8	89 (16/18)	52.9
	Resistance	19 (3/16)		13 (2/15)	
5E0I	Sensitive	41 (7/17)	63.6	50 (10/20)	54.2
	Resistance	88 (14/16)		60 (9/15)	
5E0I-FA	Sensitive	61 (11/18)	58.8	40 (8/20)	62.8
	Resistance	56 (9/16)		93 (14/15)	
5T2P-BC	Sensitive	39 (7/18)	44.1	32 (6/19)	50.0
	Resistance	50 (8/16)		73 (11/15)	
5T2P-FA	Sensitive	0(0/17)	45.5	53 (10/19)	47.0
	Resistance	94 (15/16)		40 (6/15)	
5WRE-BC	Sensitive	33 (6/18)	44.1	33 (6/18)	56.2
	Resistance	64 (9/14)		86 (12/14)	
5WRE-FA	Sensitive	10 (2/20)	40.0	70 (14/20)	67.6
	Resistance	80 (12/15)		64 (9/14)	
6WFS-BC	Sensitive	28 (5/18)	40.00	30 (6/20)	51.4
	Resistance	81 (13/16)		87 (13/15)	

Note: BC and FA are chains of each monomer. Highlighted in green are systems with at least 50% prediction for both resistance/sensitivity.

## Data Availability

The data presented in this study are available in the Supplementary Materials.

## Supplementary Materials

The following materials are available online at https://www.mdpi.com/article/10.3390/v17030332/s1: Figure S1. Distinct binding modes of CpAMs in the subunit surface, Figure S2. A model of the workflow for the prediction of CAM-resistant mutations of the HBV core protein, Figure S3. Ligand interaction of BAY41-4109 with (a) T33N- and (b) P25S-resistant mutations, Figure S4. Ligand interaction of BAY41-4109 with (a) F110I- and (b) T128I-sensitive mutations, Figure S5. Three-dimensional overlays of SBA_R01/JNJ56133679 and HAP18/Bay41-4109 and ligand interaction diagrams, Tables S1–S13. Binding free energies from docking, prime MM-GBSA and MD, Table S14. The hydrogen bonds for JNJ-56136379 and BAY41-4109 in complex with the wildtype and mutants over the simulation time.
