# Supplementary material for: Computational Approaches to Predict Hepatitis B Virus Capsid Protein Mutations That Confer Resistance to Capsid Assembly Modulators
Source: Viruses. 2025 Feb 27;17(3):332. doi: 10.3390/v17030332 (PMC11945318; doi:10.3390/v17030332)
Supplement: Supplementary file 1 [file viruses-17-00332-s001.zip › viruses-3478623-supplementary.pdf]

Computational Approaches to Predict Hepatitis B Virus Capsid Protein Mutations That Confer Resistance to Capsid Assembly Modulators

Gideon Tolufashe[1], Usha Viswanathan[1], John Kulp[1] and Ju-Tao Guo[1*]

[1]Baruch S. Blumberg Institute, 3805 Old Easton Road, Doylestown, Pennsylvania, USA.

Supplemental Information

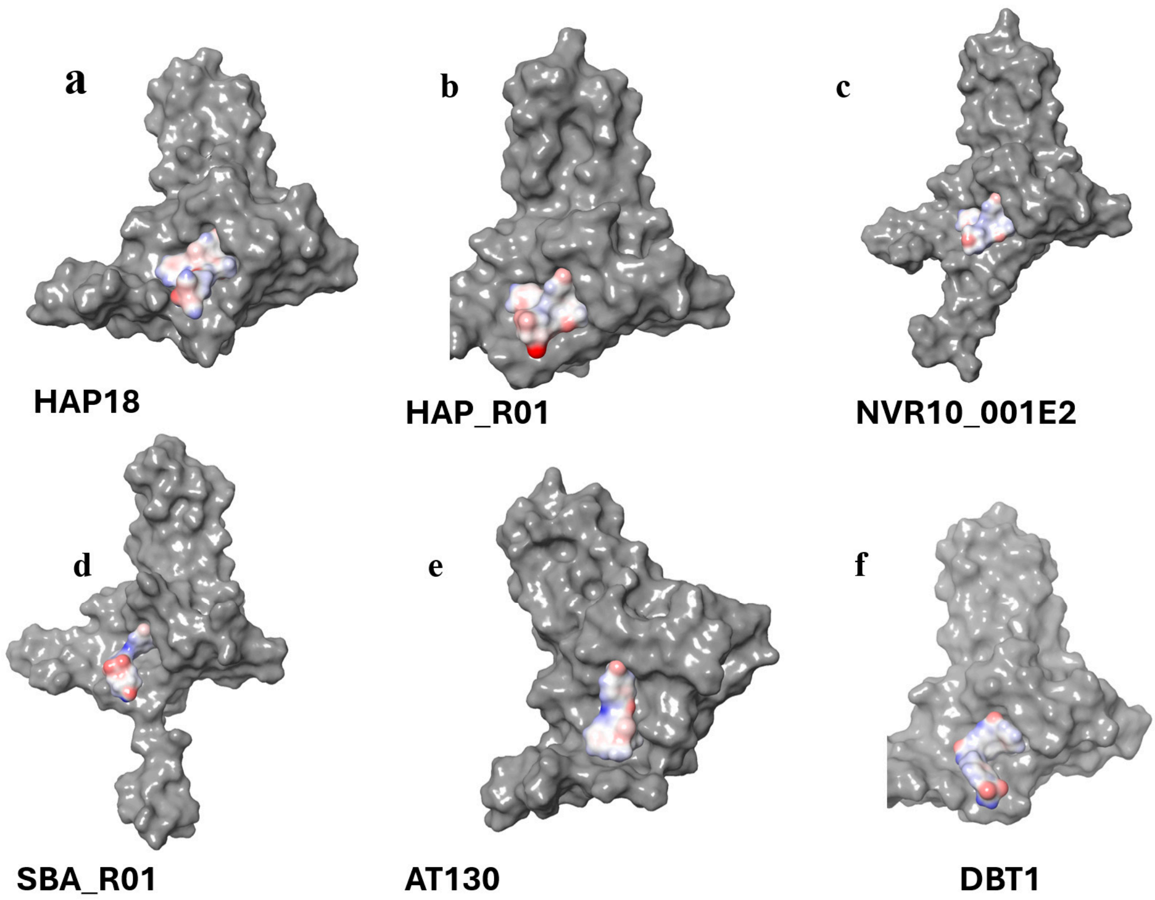

Figure S1. Distinct binding modes of CpAMs in the subunit surface. Ligand surfaces shown are, heteroaryldihydropyrimidine (HAP, a,b,c), sulfamoylbenzamide (SBA, d), phenylpropenamide (PPA, e) and dibenzothiazepine (DBT, f). HAP18, AT130 and DBT1 are bound to a capsid while HAP_R01, NVR10_001E2 and SBA_R01 are bound to CpY132A heximer.

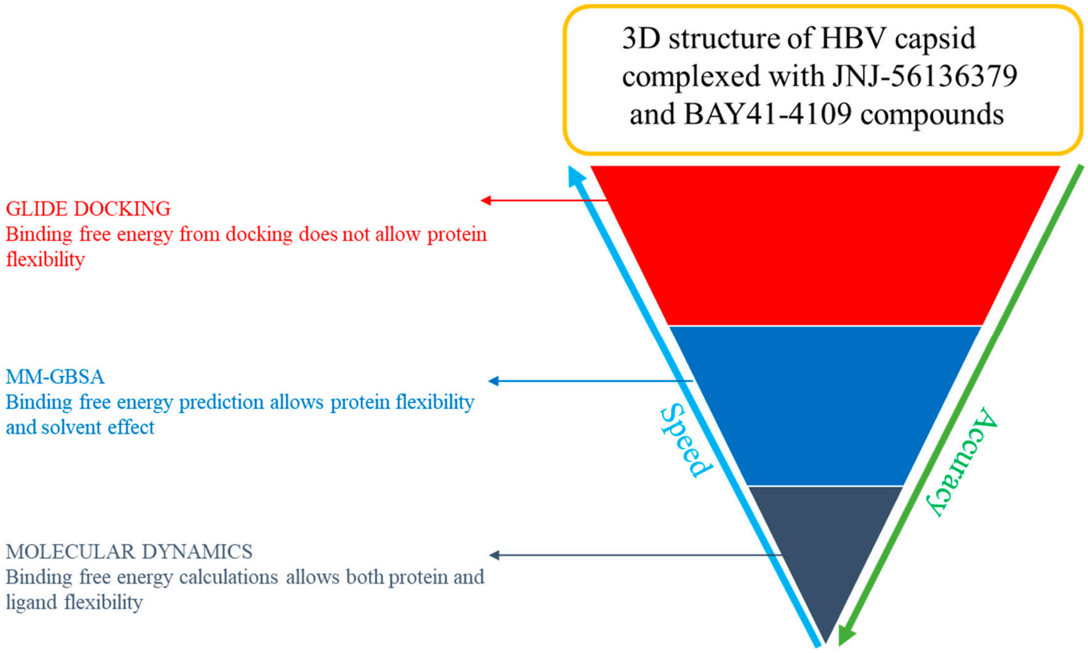

Figure S2. Modeling workflow for the prediction of CAM resistant mutations of HBV core protein.

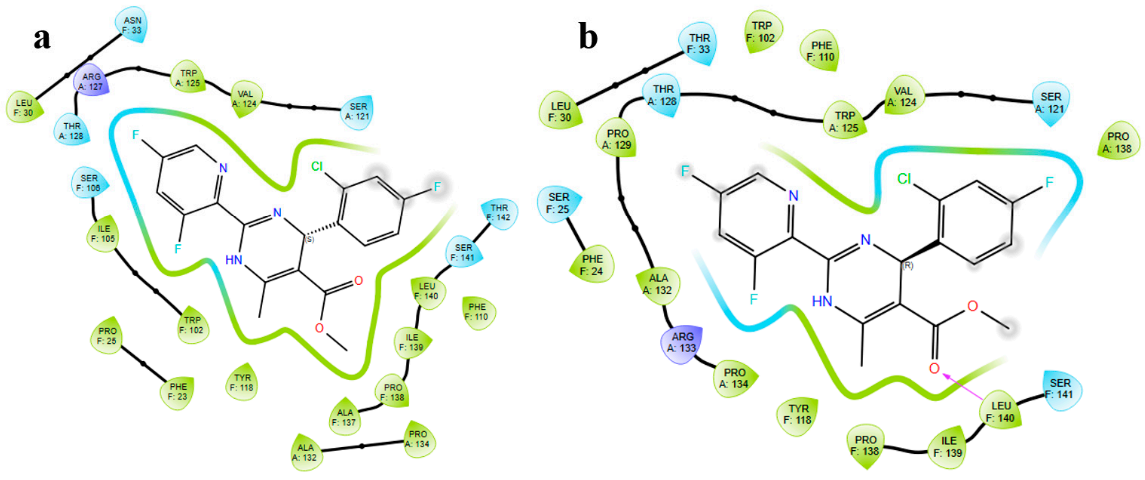

Figure S3. Ligand interaction of BAY41-4109 with (a) T33N and (b) P25S resistant mutations. HBV capsid protein (PDB ID: 5T2P-cFA).

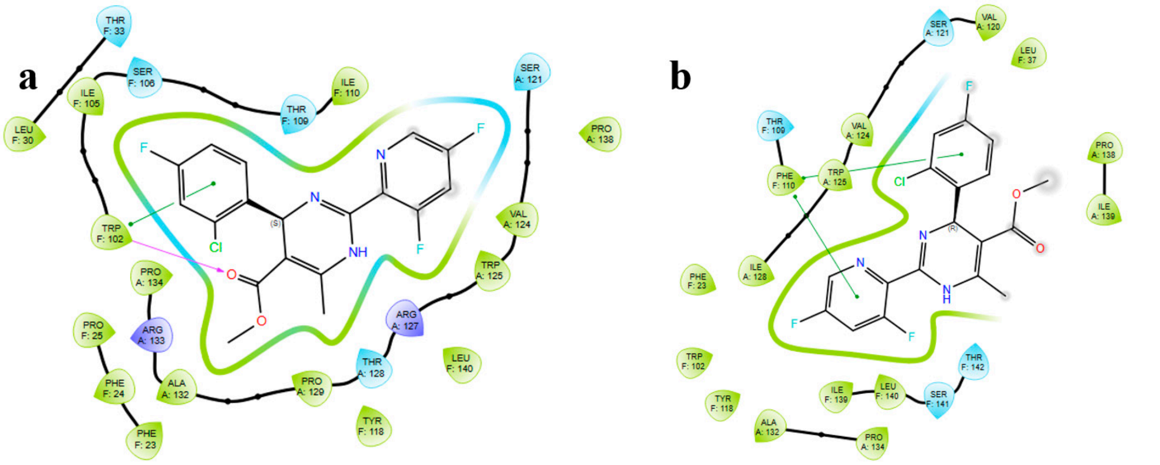

Figure S4. Ligand interaction of BAY41-4109 with (a) F110I (b) T128I sensitive mutations. Hydrogen bonds are shown in pink while green is a pi stacking using HBV capsid protein (PDB ID: 5T2P-cFA).

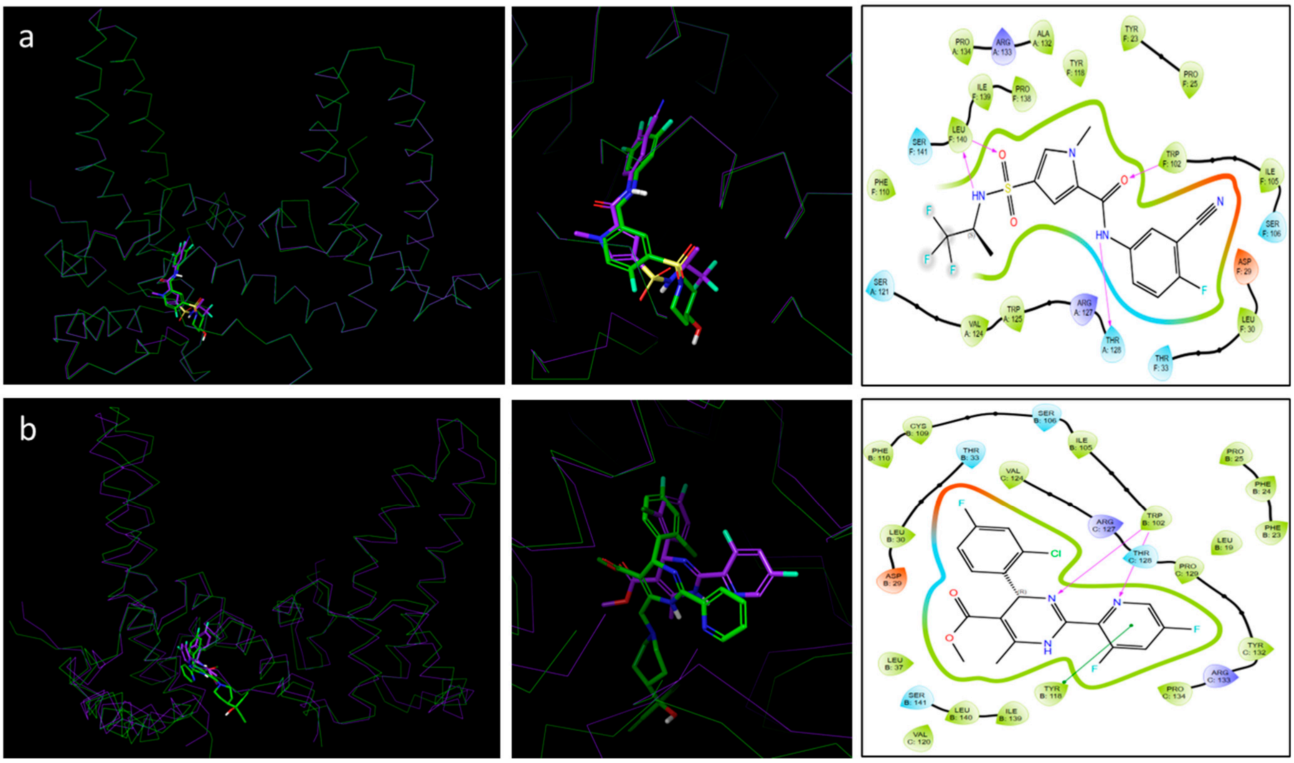

Figure S5. 3D Overlays of SBA_R01/JNJ56133679 and HAP18/Bay41-4109, and ligand interaction diagrams. Superposition of crystal structure of HBV in complex with SPA_R01, PDB:5T2P and docked conformation of it mutant with a similar chemotype, JNJ56136379, good ligand alignment [SPA_R01 (green) and JNJ56136379 (purple)] and interaction diagram of docked complex reproduced the crystal bound conformation (A) Superposition of crystal structure of HBV in complex with HAP18, PDB:5D7Y and docked conformation of it mutant with a similar chemotype,

BAY41-4109, good ligand alignment [HAP18 (green) and BAY41-4109 (purple)] and interaction diagram of docked complex reproduced the crystal bound conformation (B). Other mutants ligand interaction diagrams are provided in the supporting information, Figure S1-S9.

Table S1. Docking and MMGBSA scores for 5D7Y/HAP18 structure. Units of docking score and MMGBSA are in kcal/mol.

	Relative resistance		Docking Score		MMGBSA	
Variant	JNJ-56136379	BAY41-4109	JNJ-56136379	BAY41-4109	JNJ-56136379	BAY41-4109
WT	-	-	-6.183	-7.969	-79.72	-99.12
D29G	2.2	4.6	-6.259	-8.534	-81.48	-83.81
D29H	0.9	0.9	-6.685	-8.24	-84.79	-88.94
F110I	13	<0.5	-5.893	-7.389	-88.81	-80.32
F23Y	5.2	11	-6.62	-8.143	-80.88	-88.31
F24L	0.9	7.3	-4.478	-4.995	-79.59	-66.65
F24Y	0.6	1.6	-9.269	-6.892	-73.83	-76.81
I105L	0.5	0.5	-6.314	-4.934	-65.47	-61.29
I105T	2.7	1	-9.207	-9.307	-81.67	-92.94
I105V	1.4	1.2	-6.494	-5.039	-68.5	-80.15
L140I	1	0.09	-8.458	-8.296	-93.77	-86.21
L30F	9.3	14	-7.1	-4.995	-63.42	-61.75
L37Q	>21	3.2	-5.72	-8.381	-85.17	-89.15
P134T	<0.3	0.4	-7	-5.794	-84.14	-78.43
P25A	2.3	29	-3.702	-7.864	-78.15	-84.97
P25G	5	>26	-7.183	-8.356	-61.21	-84.42
P25S	0.4	23	-6.561	-7.889	-84.24	-86.3
R127H	3.7	>77	-10.228	-11.802	-104.19	-107.47
R133K	0.2	1.4	-10.161	-11.312	-92.06	-95.24
S106T	3	<0.2	-3.201	-7.288	-81.81	-80.55
S141P	1.4	<0.2	-6.474	-6.915	-74.16	-79.33
T109A	0.3	0.2	-6.67	-7.159	-69.35	-70.85
T109I	0.1	27	-9.33	-10.58	-73.33	-83.64
T109M	1.2	2.7	-10.583	-8.262	-90.31	-89.04
T109S	1.8	0.3	-6.386	-8.182	-89.71	-86.28
T128I	11	<0.05	-2.075	-9.433	-52.31	-94.48
T33N	85	>9.5	-3.362	-10.785	-64.84	-105.9
T33P	14	>67	-7.769	-8.812	-68.74	-83.32
T33S	2.2	2.6	-7.734	-8.22	-62.13	-94.39
V124G	>35	1.4	-5.74	-7.305	-82.43	-82.57

V124I	-	0.4	-	-11.014	-	-95.47
W125F	0.3	0.9	-3.103	-6.918	-85.39	-85.72
Y118F	6.6	6.7	-1.572	-5.602	-82.94	-64.15
Y132F	1.1	0.3	-8.75	-9.606	-84.65	-78.72
Y38F	1.4	1.1	-6.739	-8.187	-84.02	-87.48
Y38H	0.3	0.7	-8.649	-9.237	-78.5	-75.82

Table S2. Docking and MMGBSA scores using 5E0I/ NVR10-001E2 structure (Chain BC), Units of docking score and MMGBSA are in kcal/mol.

	Relative resistance		Docking Score		MMGBSA	
Variant	JNJ-56136379	BAY41-4109	JNJ-56136379	BAY41-4109	JNJ-56136379	BAY41-4109
**WT**	**-**	-	**-7.315**	**-9.468**	**-84.4**	**-97.62**
D29G	2.2	4.6	-7.682	-8.653	-83.26	-99.81
D29H	0.9	0.9	-5.353	-8.59	-88.24	-99.35
F110I	13	<0.5	-5.404	-8.261	-80.99	-97.96
F23Y	5.2	11	-6.215	-9.67	-82.1	-97.78
F24L	0.9	7.3	-5.686	-9.167	-77.02	-98.79
F24Y	0.6	1.6	-5.96	-9.529	-82.71	-99.96
I105L	0.5	0.5	-6.076	-9.193	-87.07	-102.1
I105T	2.7	1	-5.666	-8.899	-83.47	-97.32
I105V	1.4	1.2	-5.918	-7.805	-75.92	-82.83
L140I	1	0.09	-6.297	-9.268	-84.55	-95.7
L30F	9.3	14	-8.074	-9.382	-82.5	-97.54
L37Q	>21	3.2	-5.837	-8.131	-86.49	-98.19
P134T	<0.3	0.4	-7.772	-9.448	-81.21	-97.65
P25A	2.3	29	-7.196	-9.01	-82.05	-92.2
P25G	5	>26	-5.569	-7.746	-78.72	-84.62
P25S	0.4	23	-7.126	-7.675	-82.44	-90.21
R127H	3.7	>77	-6.687	-8.772	-81.7	-91.96
R133K	0.2	1.4	-5.726	-9.829	-82.84	-96.29
S106T	3	<0.2	-7.804	-9.159	-85.69	-98.85
S141P	1.4	<0.2	-7.322	-8.649	-79.67	-96.25
T109A	0.3	0.2	-7.02	-8.15	-84.78	-97.32
T109I	0.1	27	-6.422	-8.638	-85.26	-94.05
T109M	1.2	2.7	-7.4	-9.489	-81.9	-98.25
T109S	1.8	0.3	-5.221	-8.412	-85.91	-98.15
T128I	11	<0.05	-5.44	-10.395	-75.23	-98.61
T33N	85	>9.5	-7.33	-9.049	-81.65	-94.55
T33P	14	>67	-4.797	-8.418	-79.75	-80.47
T33S	2.2	2.6	-7.275	-8.659	-83.6	-94.89
V124G	>35	1.4	-6.234	-8.015	-79.12	-90.96

V124I	-	0.4	-	-8.748	-	-90.11
W125F	0.3	0.9	-7.602	-10.189	-83.45	-99
Y118F	6.6	6.7	-6.145	-9.467	-80.21	-98.86
Y132F	1.1	0.3	-7.232	-8.372	-78.67	-85.21
Y38F	1.4	1.1	-7.332	-9.492	-85.17	-96.71
Y38H	0.3	0.7	-6.088	-9.443	-79.2	-97.86

Table S3. Docking and MMGBSA scores using the 5E0I/ NVR10-001E2 structure (Chain FA)**.** Units of docking score and MMGBSA are in kcal/mol.

	Relative resistance		Docking Score		MMGBSA	
Variant	JNJ-56136379	BAY41-4109	JNJ-56136379	BAY41-4109	JNJ-56136379	BAY41-4109
WT	-	-	-7.354	-10.205	-80.73	-98.77
D29G	2.2	4.6	-6.697	-10.136	-78.48	-99.66
D29H	0.9	0.9	-7.759	-10.327	-83.47	-101.01
F110I	13	<0.5	-5.091	-9.315	-81.42	-97.08
F23Y	5.2	11	-7.54	-9.798	-77.13	-98.14
F24L	0.9	7.3	-6.648	-10.579	-81.9	-98.6
F24Y	0.6	1.6	-6.778	-9.943	-79.23	-99.13
I105L	0.5	0.5	-6.66	-8.93	-82.56	-100.35
I105T	2.7	1	-5.905	-8.977	-83.76	-98.32
I105V	1.4	1.2	-6.085	-7.43	-83.34	-91.08
L140I	1	0.09	-7.739	-9.933	-81.94	-94.59
L30F	9.3	14	-7.581	-10.084	-83.75	-93.55
L37Q	>21	3.2	-6.083	-10.138	-82.44	-97.14
P134T	<0.3	0.4	-6.922	-9.069	-82.24	-97.42
P25A	2.3	29	-5.356	-9.623	-82.8	-93.57
P25G	5	>26	-4.228	-9.479	-79.04	-90.97
P25S	0.4	23	-5.749	-9.507	-82.7	-90.16
R127H	3.7	>77	-4.672	-9.907	-76.03	-93.45
R133K	0.2	1.4	-7.596	-10.566	-81.21	-95.03
S106T	3	<0.2	-6.364	-8.719	-85.42	-102.57
S141P	1.4	<0.2	-6.105	-9.024	-73.68	-89.85
T109A	0.3	0.2	-7.174	-10.427	-82.13	-97.33
T109I	0.1	27	-7.358	-9.099	-83.39	-95.31
T109M	1.2	2.7	-5.36	-9.516	-80.04	-94.49
T109S	1.8	0.3	-6.646	-10.463	-80.18	-98.38
T128I	11	<0.05	-6.497	-11.721	-63.58	-102.25
T33N	85	>9.5	-5.922	-10.252	-72.4	-94.17
T33P	14	>67	-7.214	-10.231	-79.6	-84.22
T33S	2.2	2.6	-8.216	-10.08	-82.15	-95.74
V124G	>35	1.4	-6.311	-8.581	-72.38	-89.7
V124I	-	0.4	-	-10.516	-	-87.45

W125F	0.3	0.9	-7.26	-8.918	-82.86	-97.12
Y118F	6.6	6.7	-6.437	-10.119	-75.92	-97.87
Y132F	1.1	0.3	-6.378	-10.212	-79.51	-101.82
Y38F	1.4	1.1	-6.474	-10.139	-78.65	-99.34
Y38H	0.3	0.7	-6.192	-10.105	-78.71	-99.15

Table S4. Docking and MMGBSA scores for 5T2P/ SBA_R01 structure (Chain BC). Units of docking score and MMGBSA are in kcal/mol.

	Relative resistance		Docking Score		MMGBSA	
Variant	JNJ-56136379	BAY41-4109	JNJ-56136379	BAY41-4109	JNJ-56136379	BAY41-4109
WT	-	-	-7.288	-8.431	-79.92	-88.16
D29G	2.2	4.6	-7.682	-8.653	-71.18	-99.81
D29H	0.9	0.9	-4.997	-8.653	-83.26	-89.6
F110I	13	<0.5	-5.539	-6.974	-77.38	-85.96
F23Y	5.2	11	-6.825	-8.141	-83.7	-86.8
F24L	0.9	7.3	-6.628	-8.082	-82.39	-81.31
F24Y	0.6	1.6	-6.22	-6.683	-80.45	-80.09
I105L	0.5	0.5	-6.096	-5.31	-82.19	-82.33
I105T	2.7	1	-5.968	-8.536	-80.8	-78.66
I105V	1.4	1.2	-5.443	-8.609	-80.73	-85.99
L140I	1	0.09	-6.81	-3.036	-73.63	-81.3
L30F	9.3	14	-6.473	-8.873	-78.69	-92.07
L37Q	>21	3.2	-6.631	-8.525	-80.1	-80.66
P134T	0.3	0.4	-6.182	-8.182	-82.74	-88.8
P25A	2.3	29	-4.459	-6.274	-83.85	-82.99
P25G	5	>26	-6.553	-8.077	-76.73	-84.43
P25S	0.4	23	-7.419	-8.315	-77.46	-85.08
R127H	3.7	77	-6.972	-6.671	-79.81	-85.17
R133K	0.2	1.4	-5.839	-8.169	-75.45	-88.51
S106T	3	<0.2	-6.748	-7.585	-85.8	-86.18
S141P	1.4	0.2	-6.399	-7.601	-78.16	-84.92
T109A	0.3	0.2	-7.051	-8.544	-78.35	-86.24
T109I	0.1	27	-6.924	-8.418	-77.63	-89.58
T109M	1.2	2.7	-7.037	-7.845	-81.41	-79.32
T109S	1.8	0.3	-6.639	-8.284	-79.02	-87.04
T128I	11	0.05	-5.63	-7.38	-73.96	-69.85
T33N	85	>9.5	-7.157	-8.306	-76.49	-78.23
T33P	14	>67	-4.976	-9.225	-76.4	-84.18
T33S	2.2	2.6	-7.081	-8.448	-79.98	-89.51
V124G	>35	1.4	-5.729	-7.469	-75.88	-87.28
V124I	-	0.4	-	-5.565	-	-74.73
W125F	0.3	0.9	-6.216	-7.736	-79.57	-90.22

Y118F	6.6	6.7	-7.378	-8.555	-77.63	-81.3
Y132F	1.1	0.3	-6.267	-5.785	-73.52	-65.11
Y38F	1.4	1.1	-7.006	-8.606	-81.48	-88.79
Y38H	0.3	0.7	-6.825	-8.551	-82.05	-88.93

Table S5. Docking and MMGBSA scores for 5T2P/ SBA_R01 structure (Chain FA). Units of docking score and MMGBSA are in kcal/mol.

	Relative resistance		Docking Score		MMGBSA	
Variant	JNJ-56136379	BAY41-4109	JNJ-56136379	BAY41-4109	JNJ-56136379	BAY41-4109
WT	-	-	-7.861	-7.205	-104.68	-76.52
D29G	2.2	4.6	-7.978	-7.17	-98.34	-77.8
D29H	0.9	0.9	-7.952	-7.192	-98.01	-77.48
F110I	13	<0.5	-7.165	-8.097	-81.58	-85.3
F23Y	5.2	11	-8.355	-6.994	-100.13	-78.25
F24L	0.9	7.3	-8.029	-7.091	-98.41	-77.56
F24Y	0.6	1.6	-7.912	-7.08	-96.93	-77.13
I105L	0.5	0.5	-8.101	-7.101	-96.35	-76.1
I105T	2.7	1	-7.463	-6.824	-95.82	-76.8
I105V	1.4	1.2	-7.681	-7.207	-85.27	-75.47
L140I	1	0.09	-7.674	-6.372	-78.85	-57.42
L30F	9.3	14	-8.219	-8.033	-97.1	-75.25
L37Q	>21	3.2	-8.171	-7.159	-96.44	-76.92
P134T	<0.3	0.4	-8.088	-6.913	-93.92	-78.04
P25A	2.3	29	-7.92	-8.02	-82.05	-85
P25G	5	>26	-7.494	-6.784	-92.81	-75.04
P25S	0.4	23	-7.874	-7.022	-95.59	-78.85
R127H	3.7	>77	-7.723	-5.943	-78.22	-56.72
R133K	0.2	1.4	-8.177	-7.357	-97.35	-75.4
S106T	3	<0.2	-7.739	-8.837	-86.36	-89.77
S141P	1.4	<0.2	-7.423	-8.432	-83.15	-78.88
T109A	0.3	0.2	-8.363	-7.317	-96.94	-78.55
T109I	0.1	27	-8.611	-7.345	-98.14	-76.98
T109M	1.2	2.7	-8.537	-5.146	-100.47	-79.16
T109S	1.8	0.3	-7.993	-7.483	-102.01	-75.47
T128I	11	<0.05	-7.826	-7.535	-91.84	-66.69
T33N	85	>9.5	-7.446	-7.19	-105.11	-76.19
T33P	14	>67	-7.484	-7.353	-81.35	-74.61
T33S	2.2	2.6	-8.418	-7.116	-95.82	-75.06
V124G	>35	1.4	-8.023	-6.97	-85.04	-72.71
V124I	-	0.4	-	-5.565	-	-74.73
W125F	0.3	0.9	-8.372	-7.405	-97.08	-77.5
Y118F	6.6	6.7	-8.483	-7.583	-91.91	-79.36

Y132F	1.1	0.3	-7.316	-5.852	-83.36	-62.43
Y38F	1.4	1.1	-7.965	-6.721	-104.67	-75.71
Y38H	0.3	0.7	-8.266	-7.191	-97.33	-77.05

Table S6. Docking and MMGBSA scores for 5WRE/ HAP_R01 structure (Chain BC). Units of docking score and MMGBSA are in kcal/mol.

	Relative resistance		Docking Score		MMGBSA	
Variant	JNJ-56136379	BAY41-4109	JNJ-56136379	BAY41-4109	JNJ-56136379	BAY41-4109
WT	-	-	-6.169	-9.825	-76.51	-100.77
D29G	2.2	4.6	-5.983	-9.462	-76.82	-100.1
D29H	0.9	0.9	-2.96	-9.397	-73.65	-102.66
F110I	13	<0.5	-6.325	-8.189	-69.8	-96.39
F23Y	5.2	11	-2.262	-9.882	-68.24	-96.15
F24L	0.9	7.3	-5.696	-8.628	-79.23	-99.81
F24Y	0.6	1.6	-5.891	-9.415	-75.69	-100.63
I105L	0.5	0.5	-5.351	-9.506	-70.91	-103.51
I105T	2.7	1	-5.772	-9.452	-76.07	-100.54
I105V	1.4	1.2	-5.946	-8.157	-72.37	-86.33
L140I	1	0.09	-5.774	-9.369	-77.08	-98.11
L30F	9.3	14	-7.355	-10.443	-74.28	-97.89
P134T	>21	3.2	-5.788	-9.705	-71.42	-99.73
P25A	<0.3	0.4	-4.763	-8.853	-80.41	-93.59
P25G	2.3	29	-0.914	-8.651	-73.35	-93.02
P25S	5	>26	-5.343	-9.092	-71.9	-92.74
R127H	0.4	23	-5.983	-8.274	-71.19	-95.98
R133K	3.7	>77	-7.006	-10.599	-67.88	-99.48
S106T	0.2	1.4	-6.174	-8.671	-84.8	-101.64
S141P	3	<0.2	-5.99	-9.106	-81.32	-102.64
T109A	1.4	<0.2	-6.408	-9.575	-71.47	-99.66
T109I	0.3	0.2	-6.31	-9.216	-80.72	-97.49
T109M	0.1	27	-6.975	-9.972	-78.08	-90.39
T109S	1.2	2.7	-6.241	-8.863	-75.05	-100.52
T33N	1.8	0.3	-3.424	-6.299	-72.51	-82.29
T33P	11	<0.05	-6.612	-7.902	-75.4	-80.33
T33S	85	>9.5	-6.29	-9.146	-82.43	-105.62
V124G	14	>67	-4.781	-7.223	-67.09	-93.69
V124I	2.2	2.6	-	-7.97	-	-88.17
W125F	>35	1.4	-6.343	-8.875	-79.75	-101.01
Y118F	-	0.4	-3.359	-10.233	-46.41	-101.71
Y132F	0.3	0.9	-6.369	-7.222	-76.42	-72.81
Y38F	6.6	6.7	-6.161	-9.71	-76.72	-100.9

Table S7. Docking and MMGBSA scores for 5WRE/ HAP_R01 structure (Chain FA). Units of docking score and MMGBSA are in kcal/mol.

	Relative resistance		Docking Score		MMGBSA	
Variant	JNJ-56136379	BAY41-4109	JNJ-56136379	BAY41-4109	JNJ-56136379	BAY41-4109
	-	-	-4.246	-7.659	-77.89	-96.03
D29G	2.2	4.6	-5.135	-9.014	-67.53	-98.98
D29H	0.9	0.9	-5.115	-9.622	-79.14	-99.62
F110I	13	0.5	-5.481	-9.278	-75.12	-97.49
F23Y	5.2	11	-4.165	-9.677	-72.01	-99.38
F24L	0.9	7.3	-5.784	-9.799	-75.26	-98.5
F24Y	0.6	1.6	-6.401	-9.701	-74.44	-99.04
I105L	0.5	0.5	-6.048	-9.268	-75.41	-99.24
I105T	2.7	1	-5.866	-9.482	-77.23	-96.49
I105V	1.4	1.2	-4.826	-9.03	-75.86	-93.49
L140I	1	0.09	-3.646	-9.739	-64.36	-90.07
L30F	9.3	14	-5.664	-9.222	-77.88	-93.38
L37Q	>21	3.2	-5.959	-9.208	-76.4	-99.15
P134T	0.3	0.4	-3.707	-7.767	-73.7	-98.64
P25A	2.3	29	-4.993	-9.253	-70.51	-95.25
P25G	5	>26	-4.457	-8.688	-84.46	-93.24
P25S	0.4	23	-4.761	-8.423	-78.54	-92.37
R127H	3.7	77	-5.151	-8.84	-71.28	-88.67
R133K	0.2	1.4	-6.005	-8.984	-72.87	-94.59
S106T	3	<0.2	-5.707	-8.87	-78.12	-102.01
S141P	1.4	0.2	-5.92	-8.783	-73.9	-98.62
T109A	0.3	0.2	-6.292	-9.055	-75.48	-97.07
T109I	0.1	27	-5.628	-8.807	-77.59	-93.33
T109M	1.2	2.7	-6.164	-9.912	-70.2	-94.72
T109S	1.8	0.3	-5.867	-8.402	-70	-96.37
T128I	11	0.05	-5.732	-9.989	-77.22	-98.63
T33N	85	>9.5	-5.586	-8.325	-74.07	-93.94
T33P	14	>67	-5.649	-6.414	-71.41	-79.74
T33S	2.2	2.6	-4.885	-8.641	-78.18	-97.66
V124G	>35	1.4	-4.735	-7.466	-68.76	-90.11
V124I	-	0.4	-	-9.72	-	-101.4
W125F	0.3	0.9	-6.78	-9.292	-73.07	-98.77
Y132F	1.1	0.3	-5.063	-7.894	-59.37	-77.03
Y38F	1.4	1.1	-4.753	-9.563	-76.58	-97.72
Y38H	0.3	0.7	-6.531	-9.463	-77.1	-95.06

Table S8. Docking and MMGBSA scores for 4G93/AT-130 structure (Chain BC). Units of docking score and MMGBSA are in kcal/mol.

	Relative resistance		Docking Score		MMGBSA	
Variant	JNJ-56136379	BAY41-4109	JNJ-56136379	BAY41-4109	JNJ-56136379	BAY41-4109
WT	-	-	-4.572	-6.723	-24.34	-61.86
D29G	2.2	4.6	-5.414	-6.8	-83.67	-69.02
D29H	0.9	0.9	-5.617	-6.795	-50.85	-62.78
F110I	13	<0.5	-4.303	-7.11	-74.63	-67.95
F23Y	5.2	11	-5.106	-6.858	-69.51	-76.96
F24L	0.9	7.3	-5.005	-7.38	-78.86	-78.62
F24Y	0.6	1.6	-5.29	-6.771	-77.68	-68.98
I105L	0.5	0.5	-4.674	-6.701	-71.7	-67.99
I105T	2.7	1	-5.547	-6.505	-63.27	-94.37
I105V	1.4	1.2	-4.569	-6.75	-87.2	-91.42
L30F	9.3	14	-4.422	-7.02	-67.29	-69.9
L37Q	>21	3.2	-5.671	-6.438	-43.81	-77.8
P134T	<0.3	0.4	-4.633	-6.8	-51.85	-82.78
P25A	2.3	29	-5.013	-6.577	-68.27	-72.43
P25G	5	>26	-6.286	-6.316	-77.14	-70.25
P25S	0.4	23	-4.284	-6.811	-70.86	-82.87
R127H	3.7	>77	-4.673	-6.754	-70.15	-73.75
R133K	0.2	1.4	-5.523	-7.856	-64.84	-67.04
S106T	3	<0.2	-3.834	-7.057	-81.83	-73.01
T109A	0.3	0.2	-3.277	-6.726	-52.52	-51.9
T109I	0.1	27	-5.174	-6.604	-59.82	-74.67
T109M	1.2	2.7	-4.717	-6.529	-62.3	-70.76
T109S	1.8	0.3	-5.339	-8.306	-67.92	-72.68
T128I	11	<0.05	-5.282	-6.662	-60.53	-63.87
T33N	85	>9.5	-6.463	-7.206	-74.7	-78.92
T33P	14	>67	-6.252	-6.905	-69.46	-56.06
T33S	2.2	2.6	-6.592	-7.305	-77.17	-81.37
V124G	>35	1.4	-4.607	-6.842	-63.34	-63.91
V124I		0.4	-4.847	-7.106	-66.98	-84.6
W125F	0.3	0.9	-4.355	-6.43	-74.16	-81.34
Y118F	6.6	6.7	-6.243	-7.646	-51.52	-61.29
Y132F	1.1	0.3	-6.243	-8.043	-62.01	-76.01
Y38F	1.4	1.1	-4.997	-6.635	-71.8	-62.36
Y38H	0.3	0.7	-3.93	-6.715	-68.35	-61.55

Table S9. Docking and MMGBSA scores for 6WFS/DBT1 structure (Chain BC). Units of docking score and MMGBSA are in kcal/mol.

	Relative resistance		Docking Score		MMGBSA	
Variant	JNJ-56136379	BAY41-4109	JNJ-56136379	BAY41-4109	JNJ-56136379	BAY41-4109
WT	-	-	-8.014	-6.064	-98.29	-90.18
D29G	2.2	4.6	-7.176	-4.314	-97.73	-89.2
D29H	0.9	0.9	-7.395	-7.674	-99.44	-93.03
F110I	13	0.5	-7.786	-7.78	-86.98	-90.48
F23Y	5.2	11	-7.321	-8.764	-75.24	-78.24
F24L	0.9	7.3	-7.456	-7.398	-89.07	-89.52
F24Y	0.6	1.6	-7.251	-4.51	-96.97	-89.92
I105L	0.5	<0.5	-8.108	-5.843	-86.39	-84.32
I105T	2.7	1	-7.381	-5.796	-81.57	-91.48
I105V	1.4	1.2	-6.417	-5.704	-75.28	-79.45
L140I	1	0.09	-7.732	-8.722	-91.94	-63.34
L30F	9.3	14	-8.466	-8.015	-95.42	-89.28
L37Q	>21	3.2	-8.033	-5.975	-98.24	-89.25
P134T	<0.3	0.4	-8.408	-4.996	-97.49	-88.82
P25A	2.3	29	-7.713	-8.37	-81.88	-91.05
P25G	5	>26	-6.659	-7.679	-86.23	-81.66
P25S	0.4	23	-4.501	-7.314	-86.01	-76.86
R127H	3.7	>77	-3.272	-4.442	-99	-75.28
R133K	0.2	1.4	-8.477	-6.501	-96.88	-92.83
S106T	3	<0.2	-5.392	-4.716	-110.79	-85.29
S141P	1.4	<0.2	-6.79	-6.332	-99.93	-80.24
T109A	0.3	0.2	-7.669	-5.341	-105.45	-90.04
T109I	0.1	27	-6.542	-5.884	-98.03	-87.97
T109M	1.2	2.7	-7.172	-1.664	-97.87	-73.23
T109S	1.8	0.3	-7.904	-5.523	-99.05	-77.62
T128I	11	<0.05	-7.771	-4.998	-81.7	-76.26
T33N	85	>9.5	-6.168	-7.525	-94.5	-90.48
T33P	14	>67	-6.483	-8.64	-93.23	-76.5
T33S	2.2	2.6	-8.226	-5.91	-96.41	-64.99
V124G	>35	1.4	-6.484	-5.143	-103.42	-82.62
V124I	-	0.4	-	-7.551	-	-89.5
W125F	0.3	0.9	-8.442	-4.849	-94.68	-90.4
Y118F	6.6	6.7	-6.08	-8.409	-94.77	-85.14
Y132F	1.1	0.3	-6.867	-5.857	-98.61	-89.91
Y38F	1.4	1.1	-8.145	-4.45	-95.72	-88.62
Y38H	0.3	0.7	-7.788	-7.357	-96.92	-91.91

Table 10. Docking score for JNJ-56136379 and BAY41-4109 from 5D7Y (chain BC) and 5T2P (chain FA) models respectively representing the best predictive models for each ligand.

Position	Variant	JNJ-56136379(relative resistance)	JNJ-56136379(Docking score (kcal/mol))	BAY41-4109(relative resistance)	BAY41-4109(Docking score (kcal/mol))
	WT	-	-6.183	-	-7.205
23	F23Y	5.2	-6.62	11	-6.994
24	F24L	0.9	-4.478	7.3	-7.091
	F24Y	0.6	-9.269	1.6	-7.08
25	P25A	2.3	-3.702	29	-8.02
	P25G	5	-7.183	>26	-6.784
	P25S	0.4	-6.561	23	-7.022
29	D29G	2.2	-6.259	4.6	-7.17
	D29H	0.9	-6.685	0.9	-7.192
30	L30F	9.3	-7.1	14	-8.033
33	T33N	85	-3.362	>9.5	-7.19
	T33P	14	-7.769	>67	-7.353
	T33S	2.2	-7.734	2.6	-7.116
37	L37Q	>21	-5.72	3.2	-7.159
38	Y38F	1.4	-6.739	1.1	-6.721
	Y38H	0.3	-8.649	0.7	-7.191
105	I105L	0.5	-6.314	0.5	-7.101
	I105T	2.7	-9.207	1	-6.824
	I105V	1.4	-6.494	1.2	-7.207
106	S106T	3	-3.201	<0.2	-8.837
109	T109A	0.3	-6.67	0.2	-7.317
	T109I	0.1	-9.33	27	-7.345
	T109M	1.2	-10.583	2.7	-5.146
	T109S	1.8	-6.386	0.3	-7.483
110	F110I	13	-5.893	<0.5	-8.097
118	Y118F	6.6	-1.572	6.7	-7.583
124	V124G	>35	-5.74	1.4	-6.97
	V124I	-	-	0.4	-5.565
125	W125F	0.3	-3.103	0.9	-7.405
127	R127H	3.7	-10.228	77	-5.943
128	T128I	11	-2.075	<0.05	-7.535
132	Y132F	1.1	-8.75	0.3	-5.852
133	R133K	0.2	-10.161	1.4	-7.357
134	P134T	<0.3	-7.0	0.4	-6.913
140	L140I	1	-8.458	0.09	-6.372
141	S141P	1.4	-6.474	<0.2	-8.432

Note: Highlighted in blue/red indicate the predicted sensitivity/resistance of JNJ-56136379 and BAY41-4109 based on the docking scores. [a]Fold changes (FCs) in EC50 values compared with the HBV genotype D reference sequence.

Table 11. Binding free energy (MM/GBSA) for JNJ-56136379 and BAY41-4109 from 5E0I (chain BC) model representing the best predictive models for both ligands.

Position	Variant	JNJ-56136379 (Relative resistance)	MMGBSA (Kcal/mol)	BAY41-4109 (Relative resistance)	MMGBSA (Kcal/mol)
	WT	-	-84.4	-	-97.62
23	F23Y	5.2	-82.1	11	-97.78
24	F24L	0.9	-77.02	7.3	-98.79
	F24Y	0.6	-82.71	1.6	-99.96
25	P25A	2.3	-82.05	29	-92.20
	P25G	5	-78.72	>26	-84.62
	P25S	0.4	-82.44	23	-90.21
29	D29G	2.2	-83.26	4.6	-99.81
	D29H	0.9	-88.24	0.9	-99.35
30	L30F	9.3	-82.5	14	-97.54
33	T33N	85	-81.65	>9.5	-94.55
	T33P	14	-79.75	>67	-80.47
	T33S	2.2	-83.6	2.6	-94.89
37	L37Q	>21	-86.49	3.2	-98.19
38	Y38F	1.4	-85.17	1.1	-96.71
	Y38H	0.3	-79.2	0.7	-97.86
105	I105L	0.5	-87.07	0.5	-102.1
	I105T	2.7	-83.47	1	-97.32
	I105V	1.4	-75.92	1.2	-82.83
106	S106T	3	-85.69	<0.2	-98.85
109	T109A	0.3	-84.78	0.2	-97.32
	T109I	0.1	-85.26	27	-94.05
	T109M	1.2	-81.9	2.7	-98.25
	T109S	1.8	-85.91	0.3	-98.15
110	F110I	13	-80.99	<0.5	-97.96
118	Y118F	6.6	-80.21	6.7	-98.86
124	V124G	>35	-79.12	1.4	-90.96
	V124I	-	-	0.4	-90.11
125	W125F	0.3	-83.45	0.9	-99.00
127	R127H	3.7	-81.7	>77	-91.96
128	T128I	11	-75.23	<0.05	-98.61
132	Y132F	1.1	-78.67	0.3	-85.21
133	R133K	0.2	-82.84	1.4	-96.29
134	P134T	<0.3	-81.21	0.4	-97.65
140	L140I	1	-84.55	0.09	-95.70
141	S141P	1.4	-79.67	<0.2	-96.25

Note: Highlighted in blue/red indicate predicted sensitivity/resistance of JNJ-56136379 and BAY41-4109 based on the MM/GBSA. [a]Fold changes (FCs) in EC_{50} values compared with the HBV genotype D reference sequence.

Table 12. Calculated binding free energies and its components for JNJ-56136379 in complex with wildtype and mutants using the MM/GBSA method from MD. Binding free energy for JNJ-56136379 from 5D7Y (chain BC) model. The energy components are in kcal/mol.

Complex	ΔE_{vdw}	ΔE_{ele}	ΔG_{gas}	ΔG_{polar}	$\Delta G_{nonpolar}$	$\Delta G_{solvation}$	ΔG_{bind}	Relative resistance
JNJ-WT	-56.1±2.6	-39.8±3.3	-95.8±3.9	55.5±2.7	-7.8±0.1	47.7±2.6	-48.1±2.8	-
JNJ-T33N	-42.5±2.1	-25.9±4.6	-68.4±5.1	44.9±4.4	-5.9±0.2	39.1±4.3	-29.3±2.2	85
JNJ-T33P	-40.5±2.9	-45.3±5.5	-85.8±5.8	49.7±4.2	-5.6±0.2	44.1±4.1	-41.7±3.5	14
JNJ-T33S	-37.1±2.9	-17.3±7.2	-54.3±8.0	33.2±6.0	-5.4±0.5	27.8±5.8	-26.5±3.3	2.2
JNJ-R127H	-55.3±2.6	-38.2±3.5	-93.5±4.2	54.1±2.8	-7.7±0.2	46.3±2.7	-47.1±3.0	3.7
JNJ-Y118F	-51.7±3.7	-25.5±3.6	-77.2±5.8	47.9±3.5	-7.1±0.5	40.8±3.3	-36.4±4.1	6.6
JNJ-F110I	-55.7±2.5	-40.6±3.3	-96.2±3.9	57.0±2.6	-7.8±0.1	49.2±2.6	-47.1±2.8	13
JNJ-F23Y	-51.0±3.8	-26.8±6.8	-80.8±9.5	42.6±4.5	-6.8±0.4	35.7±4.2	-45.1±6.1	5.2
JNJ-V124G	-45.4±3.4	-30.5±3.8	-75.9±5.9	47.6±4.4	-6.4±0.5	41.2±4.0	-34.8±2.9	>35
JNJ-P25G	-43.1±3.1	-35.2±7.8	-78.4±7.8	43.9±4.8	-6.5±0.9	37.4±4.7	-40.9±4.3	5.0
JNJ-L37Q	-55.4±2.5	-38.9±3.4	-95.3±3.8	54.1±2.6	-7.9±0.1	46.2±2.6	-49.1±2.7	>21
JNJ-I105T	-42.5±2.8	-25.4±4.4	-67.9±4.9	40.3±2.9	-6.1±0.3	34.1±2.8	-33.8±3.2	2.7
JNJ-D29H	-56.4±2.6	-41.6±4.5	-97.9±5.2	56.9±3.5	-7.8±0.1	49.1±3.4	-48.8±3.1	0.9
JNJ-Y38H	-50.9±2.6	-29.9±4.6	-80.8±5.5	49.1±3.8	-7.0±0.2	42.1±3.6	-38.7±3.6	1.4
JNJ-L140I	-57.5±2.5	-31.7±4.2	-89.2±4.6	49.3±3.3	-7.8±0.2	41.5±3.2	-47.7±2.7	1.0
JNJ-I105L	-55.4±2.8	-27.8±3.9	-83.5±4.6	46.0±3.2	-7.3±0.3	38.7±3.2	-44.8±2.7	0.5
JNJ-P134T	-53.7±2.7	-44.9±5.9	-98.6±5.8	55.6±3.7	-7.7±0.2	47.9±3.6	-50.6±3.7	<0.3

Note: Where JNJ; JNJ-56136379, Highlighted in blue/red indicate predicted sensitivity/resistance of JNJ-56136379 based on the binding free energies from MD

Table 13. Calculated binding free energies and its components for BAY41-4109 in complex with wildtype and mutants using the MM/GBSA method from MD. Binding free energy for BAY41-4109 from 5D7Y (chain BC) model. The energy components are in kcal/mol.

Complex	ΔE_{vdw}	ΔE_{ele}	ΔG_{gas}	ΔG_{polar}	$\Delta G_{nonpolar}$	$\Delta G_{solvation}$	ΔG_{bind}	Relative resistance
BAY-WT	-50.4±2.2	-7.10±1.2	-62.1±2.8	17.4±0.8	-6.4±0.1	10.9±0.8	-51.2±2.7	-
BAY-T33N	-50.4±2.4	-7.3±1.5	-57.7±2.9	19.2±1.0	-6.5±0.2	12.7±1.0	-45.0±2.6	>9.5
BAY-T33P	-46.7±2.9	-2.5±1.6	-49.2±3.5	16.6±1.6	-5.9±0.2	10.7±1.5	-38.5±2.6	>67
BAY-T33S	-44.2±2.8	-4.5±1.6	-48.7±3.4	16.0±1.8	-5.7±0.3	10.4±1.6	-38.3±2.7	2.6
BAY-R127H	-54.4±2.3	-5.6±1.5	60.1±2.8	17.7±1.1	-6.5±0.1	11.2±1.1	-48.9±2.5	>77
BAY-Y118F	-31.9±2.1	-4.0±1.4	-35.9±2.5	13.9±1.2	-4.2±0.2	9.7±1.2	-26.2±2.1	6.7
BAY-F110I	-48.5±2.3	-3.5±1.3	-51.9±2.6	16.6±1.2	-6.5±0.2	10.1±1.1	-41.8±2.3	<0.5
BAY-F23Y	-44.4±2.1	-3.7±1.0	-48.1±2.3	14.6±1.0	-6.1±0.2	8.6±0.8	-39.5±2.1	11
BAY-V124G	-48.2±2.1	-2.2±1.2	-50.4±2.2	14.6±0.8	-6.1±0.1	8.5±0.8	-41.9±2.1	1.4
BAY-P25G	-54.3±2.1	-5.2±1.3	-59.5±2.5	17.8±1.0	-6.3±0.1	11.5±1.0	-48.1±2.2	>26
BAY-L37Q	-44.8±2.5	-4.5±1.8	-49.2±3.2	15.5±1.3	-6.0±0.2	9.6±1.2	-39.7±2.7	3.2
BAY-I105T	-52.4±2.3	-1.4±1.3	-53.8±2.4	16.1±1.1	-6.7±0.1	9.4±1.0	-44.3±2.4	1.0
BAY-D29H	-49.6±2.2	-9.1±1.3	-58.9±2.5	21.8±1.2	-6.5±0.2	15.2±1.2	-43.4±2.3	0.9
BAY-Y38H	-46.1±3.1	-5.6±1.4	-51.7±3.6	16.7±1.2	-6.6±0.4	10.1±1.1	-41.6±0.1	0.7
BAY-L140I	-45.2±2.0	-2.5±1.2	-47.7±2.3	11.5±0.9	-5.7±0.2	5.8±0.9	-41.9±2.2	0.09
BAY-I105L	-47.4±2.3	-4.1±1.2	-51.5±2.7	15.3±0.9	-6.4±0.2	8.9±0.9	-42.6±2.5	0.5
BAY-P134T	-52.1±2.5	-5.7±1.5	-57.9±2.9	17.7±1.1	-6.4±0.1	11.3±1.1	-46.5±2.6	0.4

Where BAY; BAY41-4109, Highlighted in red indicate the predicted resistance of BAY41-4109 based on the binding free energies from MD.

Table S14. The hydrogen bonds for JNJ-56136379 and BAY41-4109 in complex with wild type and mutants over the simulation time.

Complex	Acceptor	Donor	Occupancy (%)	Distance (Å)[a]
BA-WT	LIG286-N1	TRP102-HE1-NE1	18.7	2.9
	LIG286-F2	LEU140-H-N	9.0	2.9
	LIG286-F2	TRP102-HE1-NE1	3.3	2.9
JNJ-WT	LEU140-O	LIG286-H5-N1	82.6	2.8
	LIG286-O3	TRP102-HE1-NE1	50.9	2.9
	LIG286-O3	LEU140-H-N	26.2	2.9
BA-T33N	LIG286-N1	TRP102-HE1-NE1	17.7	2.9
	LIG286-F2	LEU140-H-N	3.9	2.9
	LIG286-F2	TRP102-HE1-NE1	3.2	2.9
JNJ-T33N	LIG286-O3	THR142-H-N	3.1	2.9
	LIG286-O3	SER141-HG-OG	2.1	2.8
BA-T33P	LIG286-O1	TRP102-HE1-NE1	0.8	2.9
JNJ-T33P	LIG286-O1	SER141-HG-OG	74.6	2.7
	LIG286-O2	THR142-H-N	48.1	2.9
	LIG286-O2	THR142-HG1-OG1	40.7	2.8
BA-T33S	LIG286-F2	THR142-H-N	0.2	2.9
JNJ-T33S	LIG286-O1	SER141-HG-OG	28.5	2.7
	LIG286-O2	SER141-HG-OG	11.4	2.8
	LIG286-O2	THR142-H-N	8.6	2.9
	LIG286-O2	THR142-HG1-OG1	8.5	2.8
	LIG286-F4	TRP102-HE1-NE1	0.3	2.9
BA-P134T	LIG286-O1	TRP102-HE1-NE1	4.0	2.9
	LIG286-O2	TRP102-HE1-NE1	2.8	2.9
JNJ-P134T	LIG286-O3	TRP102-HE1-NE1	40.4	2.9
	SER141-O	LIG286-H5-N1	12.1	2.9

Where BAY; BAY41-4109 and JNJ; JNJ-56136379
